# Modulation of Soft Magnetic Properties in Fe-Based Amorphous and Nanocrystalline Alloys for Power Transformers: A Review

**DOI:** 10.3390/ma19204269

**Published:** 2026-10-09

**Authors:** Jiashuai Ge, Xinjiang Zhang, Cailiu Yin, Zhao Lu, Bin Li

**Affiliations:** 1 Guangxi Key Laboratory of Advanced Structural Materials and Carbon Neutralization, School of Materials and Environment, Guangxi Minzu University, Nanning 530006, China; 13593521954@163.com (J.G.); 20070033@gxmzu.edu.cn (C.Y.); 2 School of Materials Science and Engineering, Guilin University of Electronic Technology, Guilin 541004, China; luzhao_gx@163.com; 3 Guangxi Nanbaote Electric Manufacturing Co., Ltd., Nanning 530001, China; 13317801388@163.com

**Keywords:** Fe-based amorphous/nanocrystalline alloys, soft magnetic properties, crystallization, nanocrystalline, magnetic cores

## Abstract

Fe-based amorphous and nanocrystalline alloys are essential core materials for high-efficiency distribution transformers, high-frequency transformers, and power electronic transformers, owing to their high saturation magnetic flux density (B_s_), low coercivity (H_c_), low core loss, and excellent high-frequency response. This review summarizes recent progress in the modulation of soft magnetic properties, covering composition design, heat treatments, structural engineering, and transformer applications. For compositional design, a central issue is how to achieve high B_s_ while retaining sufficient Glass-forming ability (GFA), and appropriate additions of metalloid and transition-metal elements provide an effective route to this balance. Conventional isothermal, stress, magnetic-field, and rapid annealing processes enable structural relaxation, nanocrystallization, and anisotropy modulation. Ultrafine nanocrystals, amorphous/nanocrystalline transitional structures, and gradient and multilayer structures further improve overall performance through grain refinement, interface engineering, and domain optimization. Key engineering challenges include continuous ribbon casting, heat-treatment uniformity, winding-induced residual stress, and practical operating conditions. Future advances require the integration of in situ characterization, multiscale modeling, and machine learning, alongside advances in continuous heat treatment and low-stress manufacturing, to facilitate wider application in transformers.

## 1. Introduction

Driven by the global low-carbon energy transition, the large-scale integration of renewable energy sources, and the increasing operating frequencies of power electronic equipment, transformer core materials are facing increasingly demanding performance requirements. Conventional distribution transformers are still required to achieve high efficiency and low loss at grid frequency [1]. In contrast, medium- and high-frequency transformers for renewable-energy conversion, solid-state transformers, and high-frequency power converters are moving toward higher operating frequencies, greater compactness, and higher power density [2]. These trends place more stringent demands on core materials, which must maintain low loss and high permeability over a wide frequency range while providing sufficiently high saturation magnetic flux density (B_s_) to support further increases in power density. Conventional silicon steel, despite its mature manufacturing technology and high B_s_, suffers from rapidly increasing eddy-current loss under high-frequency and nonsinusoidal excitation [3]. It therefore becomes less suitable for modern power electronic converters operating at elevated frequencies. Ferrites exhibit high electrical resistivity (ρ) and are well suited to frequencies above several tens of kilohertz [4]. However, their relatively low B_s_ limits the achievable power density and degree of miniaturization. In this context, Fe-based amorphous and nanocrystalline alloys, owing to their distinctive structural characteristics and favorable magnetic properties, represent promising core materials for high-efficiency distribution transformers and medium- to high-frequency power electronic transformers [5].

Fe-based amorphous alloys are typically produced by rapid solidification from the melt, resulting in an atomic structure characterized by long-range disorder and short-range order [6]. The absence of long-range crystalline order suppresses magnetocrystalline anisotropy and eliminates grain boundaries and dislocations that would otherwise act as domain-wall pinning sites [7]. In addition, the high electrical ρ of these alloys, together with their thin-ribbon geometry, effectively suppresses high-frequency eddy-current loss [8]. These features provide the physical basis for their low-loss behavior. Controlled crystallization can then be used to produce finely dispersed nanocrystals [9]. Exchange coupling among adjacent nanocrystals spatially averages the randomly oriented local magnetocrystalline anisotropy [10,11,12], thereby markedly reducing the effective magnetic anisotropy and coercivity (H_c_) while maintaining high permeability and a high operating flux density [13]. The combination of an amorphous matrix and exchange-coupled nanocrystals underpins the application potential of Fe-based amorphous and nanocrystalline alloys in high-efficiency distribution transformers, medium- and high-frequency transformers, and renewable-energy power electronic systems [14].

**Figure 1:**
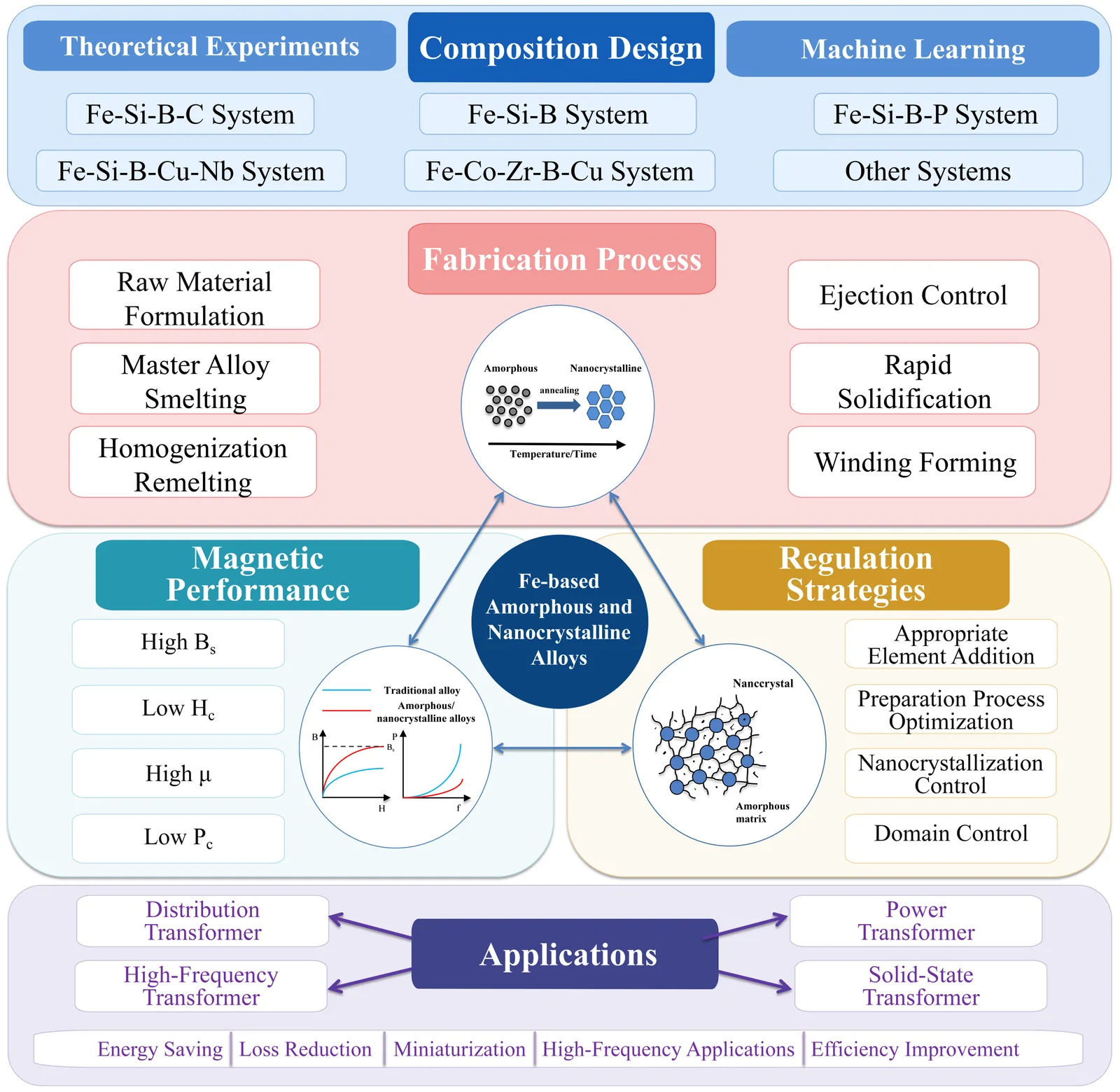
Research focuses and transformer application scenarios of Fe-based amorphous/nanocrystalline alloys.

The soft magnetic properties of Fe-based amorphous and nanocrystalline alloys are governed by multiple interdependent factors rather than by a single structural parameter [15]. These factors include alloy composition, local atomic structure, heat-treatment conditions, and core-manufacturing processes [16,17,18]. Compositional design therefore requires balancing B_s_, Glass-forming ability (GFA), thermal stability, and control over nanocrystallization. After crystallization, grain size, nanocrystalline volume fraction, the residual amorphous phase, interfacial segregation, and magnetic-domain structure jointly determine the effective magnetic anisotropy and domain evolution [19,20]. More importantly, translating favorable material-level magnetic properties into device-level performance requires a sequence of engineering processes, including ribbon casting, slitting, winding, heat treatment, core shaping, assembly, and insulation processing. The resulting cores must also maintain their performance under application-relevant operating conditions, including nonsinusoidal excitation, temperature rise, and mechanical stress [21,22]. These engineering factors can substantially degrade the performance achieved at the material level [23]. A key challenge is therefore to establish clear links among atomic-scale structure, nanocrystalline microstructure, core-manufacturing processes, and performance under practical operating conditions, as illustrated in Figure 1.

This review presents a technology roadmap for transformer applications, focusing on Fe-based amorphous and nanocrystalline alloys. It begins with fundamental issues including high-frequency loss, B_s_, and random anisotropy, and examines the trade-offs among key soft magnetic properties. It then discusses how composition design, heat treatment, and amorphous/nanocrystalline composite structures affect magnetic performance, while comparing the strengths and limitations of different modulation strategies. The review also considers how practical transformer operating conditions and manufacturing processes, including ribbon casting, winding, and impregnation, influence the transfer of material-level properties to core-level performance. In this way, material optimization is linked more directly to the engineering requirements of transformer applications. Finally, future research priorities are identified to guide alloy design, process development, and performance optimization for transformer applications.

## 2. Physical Basis of Soft Magnetic Properties

### 2.1. High-Frequency Loss Advantage and B_s_ Ceiling

The high-frequency soft magnetic performance of Fe-based amorphous/nanocrystalline alloys is closely related to the long-range disorder of their atomic structures. This structural characteristic provides two important advantages: low magnetocrystalline anisotropy and high electrical ρ. However, it can also impose an upper limit on the B_s_ because the Fe magnetic moment density is reduced and exchange interactions become less favorable [24]. Balancing these effects is therefore a key challenge in optimizing the soft magnetic performance of these materials [25]. Maximizing B_s_ while maintaining low loss at high frequencies has consequently become a major research focus. In recent years, research on this topic has progressed from macroscopic loss characterization to electronic-structure analysis, although the physical origin of this trade-off is still being clarified.

**Figure 2:**
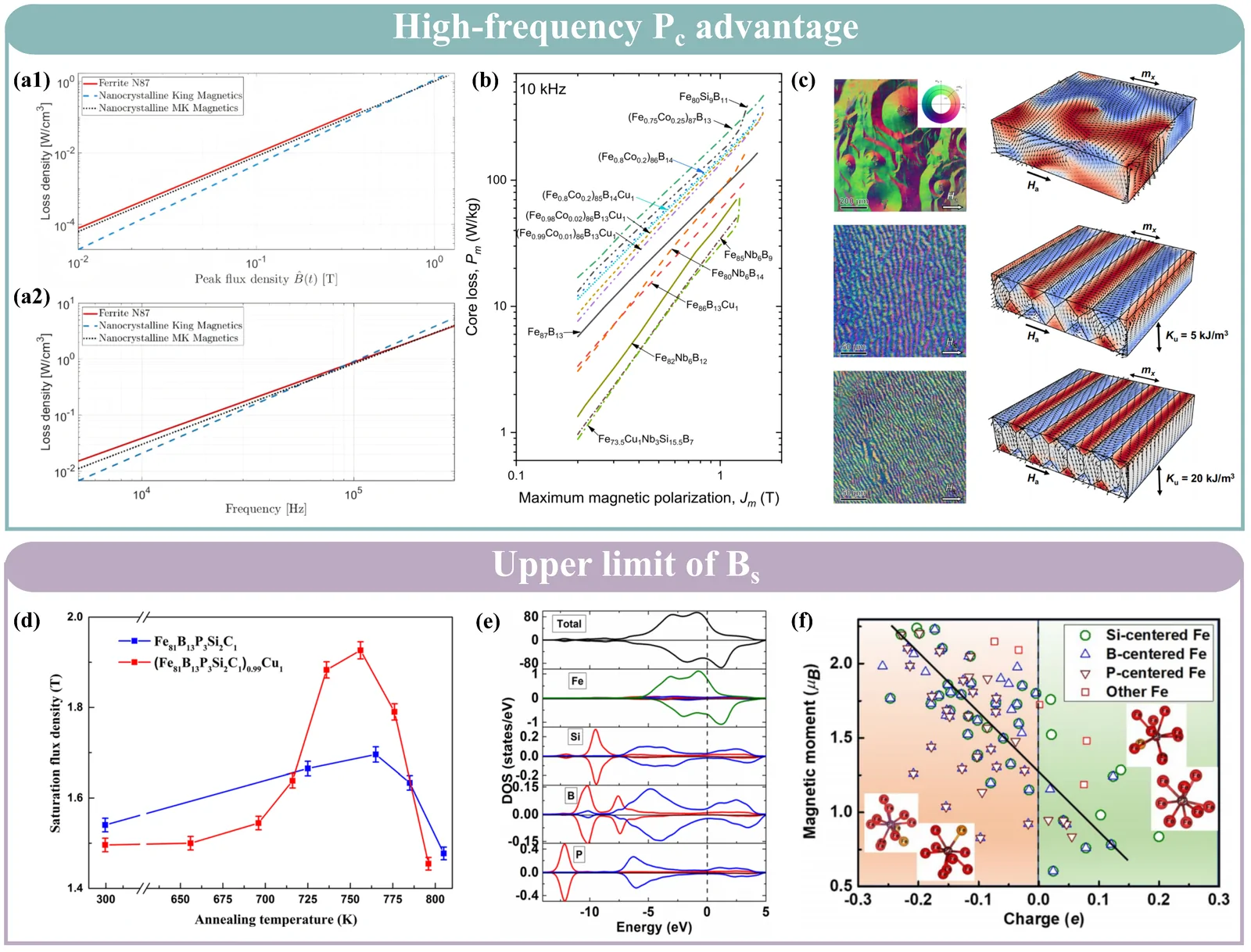
Physical basis for high-frequency low-loss behavior and B_s_ in Fe-based amorphous/nanocrystalline alloys. (**a1**,**a2**) Comparison of loss density between Fe-based amorphous/nanocrystalline materials and ferrites at different peak magnetic flux densities and excitation frequencies [26]. (**b**) Core loss of various Fe-based amorphous/nanocrystalline materials as a function of maximum magnetic polarization at 10 kHz [27]. (**c**) Evolution of magnetic domain structures in Fe-rich soft magnetic ribbons with different crystalline volume fractions [28]. (**d**) B_s_ of FeSiBPC(Cu) amorphous alloys as a function of annealing temperature [31]. (**e**,**f**) Correlation between atomic magnetic moments and electronic charge, together with calculated total and partial densities of states for FeSiBP metallic glass [32].

The high-frequency low-loss behavior of amorphous/nanocrystalline materials arises mainly from two factors. First, the long-range disorder of the amorphous precursor, the thin-ribbon geometry, and the exchange-averaging effect induced by nanocrystallization collectively help reduce magnetic anisotropy and dynamic losses. Second, nanocrystalline materials exhibit higher saturation magnetic flux densities than ferrites, enabling higher operating flux densities while maintaining low core loss at medium-to-high frequencies. Somkun et al. [26] confirmed this behavior by comparing the loss characteristics of two nanocrystalline cores with those of N87 ferrite. The nanocrystalline cores exhibited lower loss densities than N87 ferrite at 20 kHz over the flux-density range of 0.01–1.20 T (Figure 2(a1)), and this advantage persisted over 5–200 kHz at 0.30 T (Figure 2(a2)). Under identical conditions of 20 kHz and 0.30 T, the loss densities of N87 ferrite and the two nanocrystalline cores were approximately 0.100, 0.081, and 0.065 W/cm^3^, corresponding to B_s_ values of 0.39, 1.23, and 1.25 T, respectively, demonstrating that nanocrystalline materials can operate at higher flux densities while maintaining low loss at medium frequencies. However, this comparison should be regarded only as an illustrative benchmark and should not be generalized to other material systems. In practical applications, the measured core loss depends not only on the intrinsic magnetic properties of the material but also on factors such as core geometry, ribbon thickness, excitation waveform, and operating temperature. These results further show that the low-loss advantage of nanocrystalline materials remains significant under the combined conditions of elevated frequency and high operating flux density.

Notably, high-frequency core loss is not simply an extension of low-frequency hysteresis loss, but results from the combined contributions of hysteresis loss, classical eddy-current loss, and excess loss associated with domain-wall dynamics. Huang et al. [27] compared the core losses of amorphous/nanocrystalline alloys with different saturation magnetostriction coefficients (λ_s_) and found that alloys with lower λ_s_ exhibited lower core loss at 10 kHz (Figure 2b). Loss decomposition further showed that the excess loss increased with increasing λ_s_, suggesting that magnetostriction-related damping of domain-wall motion plays an important role in high-frequency loss. Domain-engineering studies also showed that weak perpendicular anisotropy induced by moderate nanocrystallization can transform wide [28], curved domains into stripe domains (Figure 2c), thereby suppressing large-amplitude domain-wall displacement and promoting magnetization rotation. However, with a further increase in perpendicular anisotropy, the domain structure continues to become finer, while magnetization rotation is increasingly restricted, causing the total loss to rise again.

High-frequency loss does not decrease monotonically with increasing degree of nanocrystallization. Instead, an optimal crystallization window exists, requiring a balance among the nanocrystalline volume fraction, residual amorphous matrix, internal stress, and domain structure. Related micromagnetic simulations suggest that the minimum in high-frequency loss mainly arises from the weak perpendicular magnetic anisotropy induced by partial nanocrystallization and the resulting changes in magnetization dynamics, rather than from a Bloch–Néel domain-wall transition or simple exchange-coupling percolation [11,29]. Moderate nanocrystallization suppresses irreversible domain-wall motion and excess loss, whereas further crystallization enhances magnetic anisotropy and restricts magnetization rotation. Magnetostriction-mediated domain-wall damping also contributes to dynamic dissipation at high frequencies. Overall, strategies for regulating high-frequency loss have gradually evolved from single-composition optimization toward multiscale synergistic control of structure, magnetic domains, and dynamic magnetization behavior. Nevertheless, the dynamic competition between domain-wall pinning and magnetization rotation at high frequencies remains to be clarified in greater depth.

However, the advantage of low loss at high frequencies is accompanied by limited improvement in B_s_, and B_s_ does not increase monotonically with increasing Fe content. Shi et al. [30] reported that Fe_83–85_(B,C,Si)_14–16_P_1_ amorphous alloys exhibited B_s_ values of 1.61–1.71 T after annealing, whereas a further increase in Fe content did not result in a continuous increase in room-temperature B_s_. Jiang et al. [31] introduced 1 at.% Cu into Fe_81_B_13_P_3_Si_2_C_1_ alloys and induced the precipitation of fine α-Fe nanocrystals through annealing, thereby increasing the B_s_ of (Fe81B13P3Si2C1)0.99Cu1 to 1.92 T (Figure 2d). With further increases in annealing temperature, however, coarsening of the α-Fe grains and the formation of unfavorable Fe–B phases led to a decrease in B_s_. These results indicate that further enhancement of B_s_ is governed not only by Fe content, but also by the extent of precipitation of high-moment phases and the stability of the microstructure. From the perspective of electronic structure [32], charge transfer between Fe and metalloid elements, together with p–d orbital hybridization, regulates the local magnetic moments. In particular, Fe atoms adjacent to P possess relatively large magnetic moments, whereas B exhibits negative spin polarization (Figure 2e,f). This electronic-scale understanding points to possible compositional spaces for further increasing B_s_, although the translation of theoretical predictions into practical alloy development remains constrained by both GFA and thermal stability.

### 2.2. Random Anisotropy in Nanocrystalline Alloys

The physical understanding of the soft magnetic properties of Fe-based nanocrystalline alloys has progressed from empirical optimization to theoretical interpretation. Among the theoretical approaches developed in this field, the random anisotropy model has been systematically introduced into studies of nanocrystalline soft magnetic alloys and has become an important theoretical basis for understanding their magnetic softening behavior [33]. It remains one of the core analytical frameworks for explaining the excellent soft magnetic properties of Fe-based nanocrystalline alloys [34].

**Figure 3:**
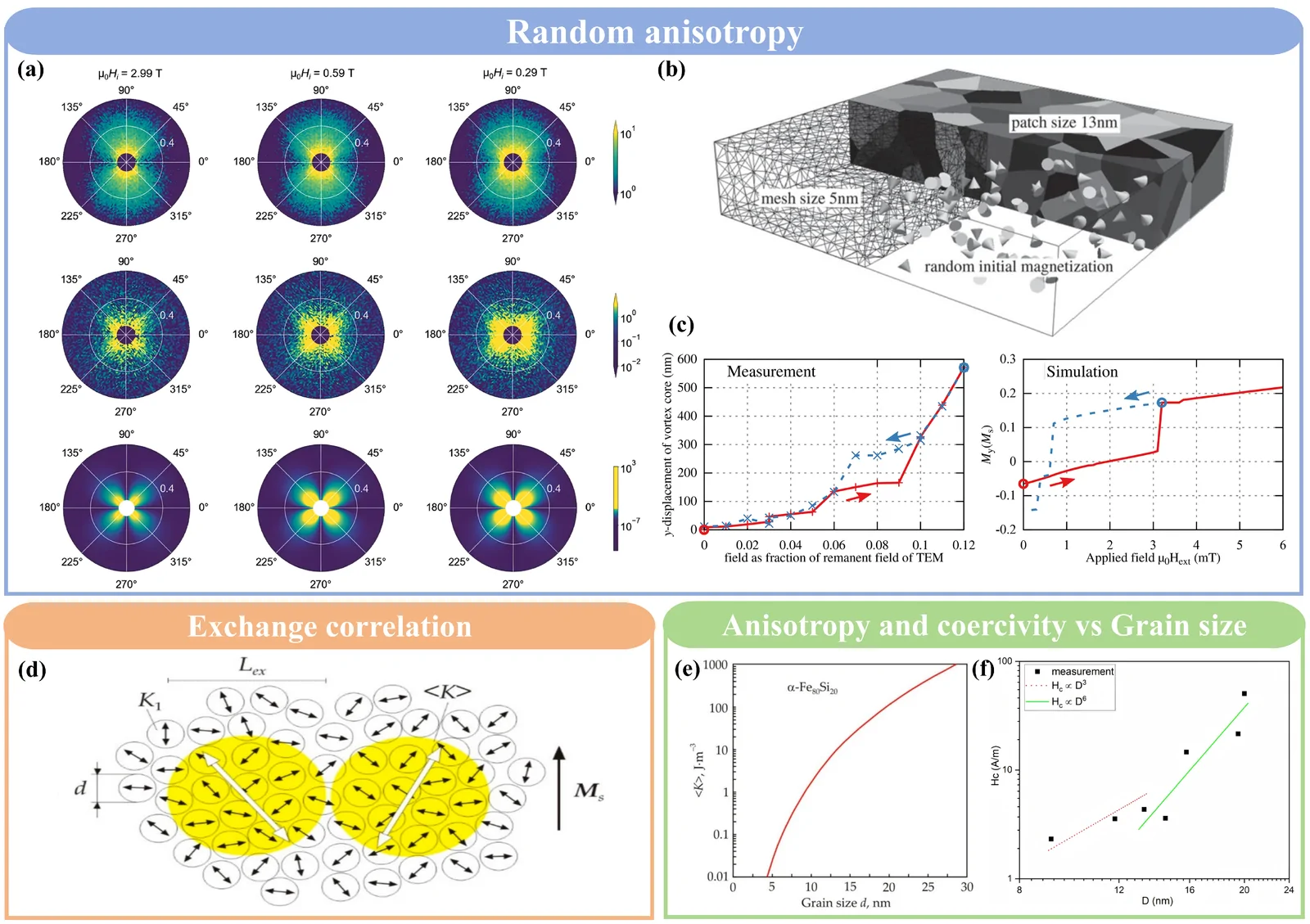
Selected experimental and theoretical studies of random anisotropy in nanocrystalline alloys. (**a**) Two-dimensional small-angle neutron scattering patterns of FeNiB nanocrystalline alloys at internal magnetic fields of μ_0_H_i_ = 2.99, 0.59, and 0.29 T, showing a characteristic four-lobed clover-leaf-like angular anisotropy [35]. (**b**) Micromagnetic model of random local anisotropy [38]. (**c**) Experimental and simulated magnetization responses in the presence of random anisotropy, arrows indicate the field-sweep direction, and dashed curves represent the return process [38]. (**d**) Schematic illustration of the magnetic structure of nanocrystalline soft magnetic alloys [40]. (**e**) Dependence of the effective magnetic anisotropy constant of α-Fe_80_Si_20_ on grain size within the random anisotropy model [40]. (**f**) H_c_ of FeSiBCuNb alloys as a function of average grain size [41].

The random anisotropy behavior of nanocrystalline alloys arises primarily from the combined effects of randomly oriented easy magnetization axes and intergranular exchange coupling. When the grain size is smaller than the exchange length L0, the magnetic moments of adjacent grains cannot independently align along their respective local easy axes. Instead, they align cooperatively within the exchange-correlation volume, causing the local magnetocrystalline anisotropies of different grains to be averaged out and thereby significantly reducing the effective anisotropy and H_c_. Bersweiler et al. [35] measured an average grain size of approximately 7 ± 1 nm and an exchange length of approximately 50 nm in (Fe_0.7_Ni_0.3_)_86_B_14_, and observed a four-leaf-clover-like magnetic scattering anisotropy that became more pronounced as the magnetic field decreased (Figure 3a). This behavior mainly originates from longitudinal magnetization jumps at the nanocrystal/amorphous-matrix interfaces and spin misalignment induced by dipolar stray fields.

Using a polydisperse grain model with randomly oriented easy axes, Bolyachkin et al. [36] further showed that the power-law exponent relating H_c_ to the average grain size gradually decreases from 6 in an ideal monodisperse system to approximately 3 as the grain size distribution broadens. This behavior can be understood by the fact that the D6 relationship is derived under ideal exchange-averaging conditions, in which all grains satisfy D < L0 and have a uniform size. In real materials, however, larger grains make stronger local contributions to random anisotropy and progressively reduce the overall efficiency of exchange averaging, thereby weakening the dependence of H_c_ on the average grain size. The model further shows that the geometric standard deviation σD of the grain size distribution is an important parameter governing this scaling transition. As σD increases, the effective anisotropy increases, resulting in a transition from D6 scaling toward D3 behavior [37]. Therefore, the soft magnetic properties of real nanocrystalline alloys are jointly governed by the grain size and the width of its distribution through their influence on the efficiency of random anisotropy averaging. Simply reducing the average grain size does not guarantee the theoretical D6 magnetic softening behavior; abnormally large grains and broadening of the grain size distribution must also be controlled.

The random anisotropy micromagnetic model further shows [38] that different local regions contain randomly oriented easy axes and initial magnetization directions (Figure 3b). Such spatial disorder gives rise to local magnetization pinning and leads to nonlinear magnetization responses and pronounced hysteresis under an external magnetic field (Figure 3c), directly illustrating the influence of random anisotropy on nanoscale magnetization processes. The statistical averaging of random anisotropy is also strongly dependent on the magnetic field, essentially because the external field changes the relationship between the magnetic correlation length and the structural correlation scale. At low magnetic fields, the local random easy axes strongly perturb the magnetization direction, and the magnetic correlation regions expand anisotropically. As the external field increases, exchange interactions increasingly dominate collective magnetization over larger length scales, and the magnetic correlation volume gradually becomes three-dimensionally isotropic. Near saturation, the deviation of the magnetic moments from the external-field direction decreases further. Ilin et al. [39] performed correlated magnetic measurements on three Fe-Cu-Nb-Si-B amorphous precursors and found that the decay of magnetization deviation followed H^−1^, H^−1/2^, and H^−2^ scaling relationships with increasing magnetic field strength, corresponding to low-field two-dimensional anisotropic correlation, intermediate-field three-dimensional isotropic correlation, and high-field near-saturation states, respectively. Therefore, random anisotropy should not be regarded as a fixed form of local disorder, but rather as a statistical state of magnetic correlations that evolves with the external field.

The effects of random anisotropy are ultimately manifested in the strong dependence of the effective anisotropy constant and H_c_ on grain size. Under ideal exchange-averaging conditions, finer grains allow a larger number of grains with randomly oriented easy axes to participate in the averaging process within a given exchange-correlation volume, resulting in a rapid decrease in the effective anisotropy. Tsepelev et al. [40] systematically examined the relationship between the effective anisotropy constant and the local magnetocrystalline anisotropy constant, while Figure 3d presents a schematic illustration of the magnetic structure of nanocrystalline soft magnetic alloys. Taking Fe_80_Si_20_ as an example, when the grain size is 10 nm, the effective anisotropy constant is approximately 2000 times lower than the local magnetocrystalline anisotropy constant (Figure 3e), clearly demonstrating the high efficiency of random anisotropy averaging. This grain-size effect has also been verified under practical processing conditions. Yao et al. [41] obtained grains of approximately 9.1–20 nm after laser annealing of Fe_72_Si_16_B_7_Cu_1_Nb_4_ and found that H_c_ approximately followed the D6 relationship in regions containing larger grains, while gradually approaching the D3 relationship in regions containing smaller grains (Figure 3f). Therefore, the soft magnetic properties of real nanocrystalline alloys depend on the combined effects of grain size and grain size distribution on the efficiency of random anisotropy averaging.

### 2.3. Soft Magnetic Property Trade-Offs

The soft magnetic properties of Fe-based amorphous/nanocrystalline alloys are not independently tunable in isolation; rather, they are intrinsically coupled through multiple physical correlations. A deeper understanding of these interdependencies is therefore essential for rational property tailoring [42]. Accordingly, the research focus in this field has gradually shifted from maximizing individual properties toward achieving a multivariable balance among high B_s_, low H_c_, high ρ, and a wide annealing window [43].

The inverse relationship between high Fe content and GFA represents one of the most critical constraints in this material system. Increasing the content of ferromagnetic elements such as Fe generally favors a higher B_s_, but at the same time narrows the compositional space available for glass-forming elements such as B, Si, and P, thereby reducing GFA and thermal stability. Consequently, a high B_s_ cannot be optimized independently of the stability of the amorphous precursor. Wang et al. [44] subjected Fe_83_Cu_1_Nb_3_Si_4_B_9_ alloys to deep supercooling treatment, which reduced atomic mobility associated with crystallization and promoted the formation of strongly bonded short-range packing motifs and icosahedral or icosahedral-like structures, thereby enhancing the stability of the amorphous phase. After annealing, the alloy achieved a B_s_ of 1.71 T, an H_c_ of 5.0 A/m, and a relatively wide crystallization temperature window. To address such multiple property constraints, Mo et al. [45] further used B_s_, crystallization temperature (T_x_), and maximum casting diameter (D_max_) as optimization objectives and applied ensemble learning combined with NSGA-III optimization to the Fe-B-Si-P-C system, obtaining 15 sets of Pareto-optimal compositions. These results suggest that magnetic properties, thermal stability, and GFA are better optimized through multi-objective trade-offs rather than by pursuing the extreme value of a single property.

**Figure 4:**
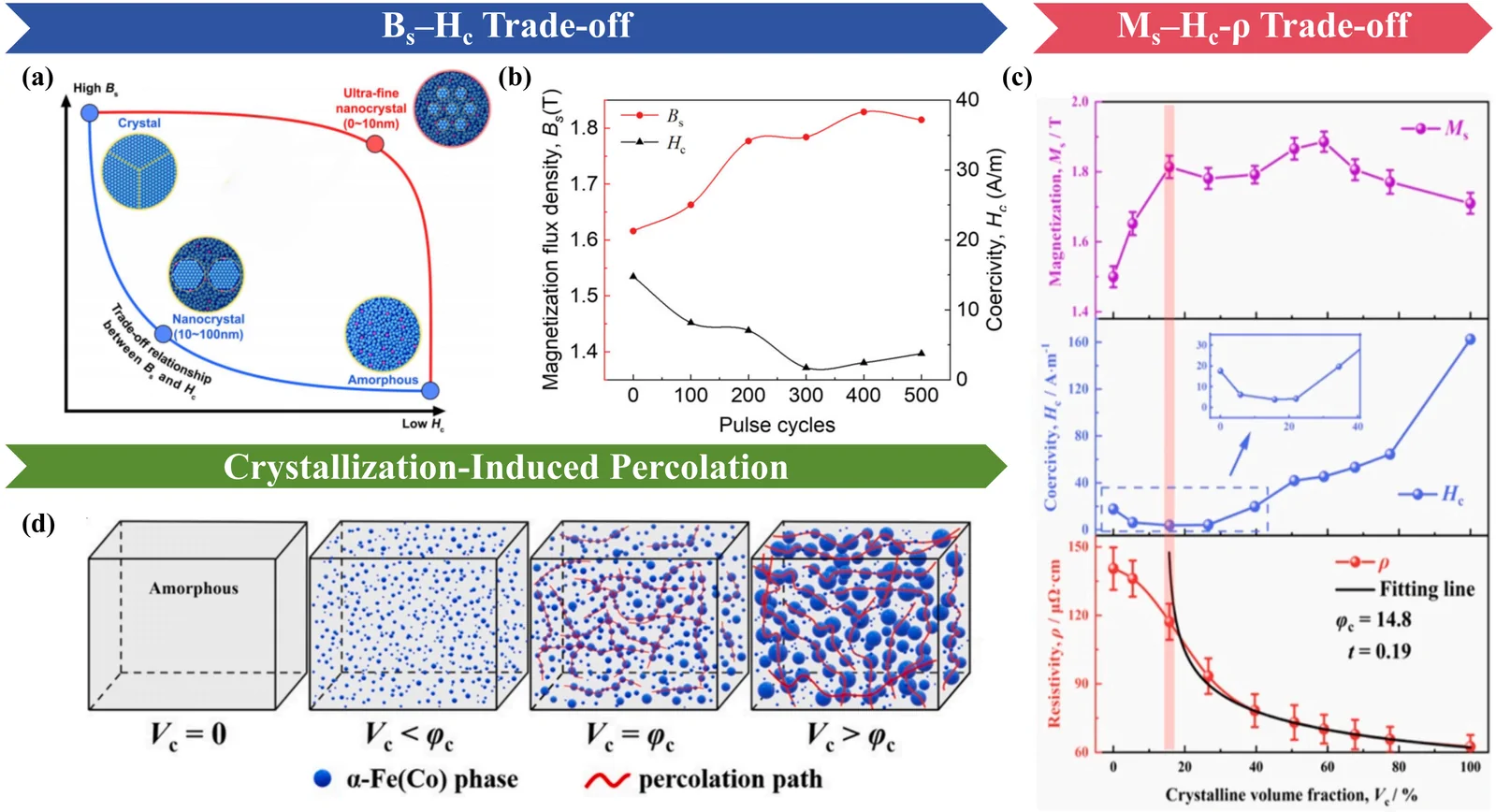
Multi-property trade-offs in amorphous/nanocrystalline soft magnetic materials. (**a**) Schematic illustration of the trade-off between B_s_ and H_c_ in different structural states [47]. (**b**) Variations in B_s_ and H_c_ during pulse treatment [48]. (**c**) Trade-offs among M_s_, H_c_, and ρ as a function of crystalline volume fraction [50]. (**d**) Schematic illustration of the crystallization-induced electrical percolation mechanism [50].

Once the composition is fixed, the interdependence among GFA, B_s_, and H_c_ is further manifested during the subsequent crystallization process. Metalloid elements affect not only the stability of the amorphous structure but also the nucleation and growth of nanocrystals; therefore, variations in their contents can simultaneously alter the fraction of magnetic atoms and the grain size. Kang et al. [46] investigated Fe_80_Si_7_B_12−x_P_x_Cu_1_ alloys and found that P increases the degree of structural disorder in the amorphous state and improves GFA, while also promoting competitive growth of α-Fe(Si) along different crystallographic orientations. When x = 4, B_s_ reached 1.75 T, whereas at x = 8, H_c_ decreased to 4.61 A/m and the grain size was refined from 61.36 nm in the P-free alloy to 21.24 nm, corresponding to improvements of approximately 3.0% in B_s_ and 75.3% in H_c_, respectively. Previous studies have shown that a pronounced trade-off exists between B_s_ and H_c_ in Fe-based soft magnetic alloys [47]. As illustrated in Figure 4a, crystalline materials generally exhibit higher B_s_ but also higher H_c_, whereas amorphous materials possess lower H_c_ at the expense of reduced B_s_. Conventional nanocrystallization can partially balance these two properties, while further refinement to ultrafine nanocrystals is expected to significantly reduce H_c_ while maintaining a high B_s_, thereby overcoming the conventional B_s_–H_c_ trade-off. During pulse treatment of Fe_83.3_Si_4_B_8_P_4_Cu_0.7_ amorphous alloys [48], B_s_ gradually increased from 1.61 T to a maximum of 1.83 T, whereas H_c_ decreased from 14.7 A/m to a minimum of 1.6 A/m (Figure 4b). However, the optimal values of B_s_ and H_c_ occurred at 400 and 300 pulse cycles, respectively, indicating that the increase in B_s_ and the reduction in H_c_ are not fully synchronized. An optimal balance between the degree of crystallization and magnetic softening is therefore still required. Thus, achieving a favorable combination of high B_s_ and low H_c_ depends not only on composition selection but also on precise control of nanocrystal size and uniformity through the crystallization pathway.

GFA and strong ferromagnetism are not intrinsically incompatible, and a high GFA does not necessarily require a significant sacrifice in magnetic performance. Although a certain trade-off between them is often observed in conventional composition design, this relationship is not determined by GFA itself, but primarily by how the local short-range order induced by alloying elements simultaneously affects glass stability and magnetic exchange interactions. Huang et al. [49] used the difference between the calculated ideal saturation magnetic flux density (B_s_,cal) and the measured B_s_, i.e., B_s_,cal − B_s_, to characterize the degree of ferromagnetic weakening relative to the ideal calculated value. They found that, in the Mo-, Nb-, and Ga-containing systems, the reduced glass transition temperature (Trg) decreased with increasing B_s_,cal − B_s_, indicating that higher GFA can coexist with stronger ferromagnetism. In contrast, the Tb- and Co-containing systems exhibited magnetic weakening with increasing GFA, whereas no clear correlation was observed in the Ni-containing system. Structural analyses suggested that solute-centered short-range order formed by Mo, Nb, and Ga can stabilize the glass structure while simultaneously adjusting the nearest-neighbor Fe–Fe distance and enhancing exchange interactions. By contrast, stable clusters formed by Tb and Co may reduce the relevant exchange strength. Therefore, the assumption that improving GFA necessarily weakens ferromagnetism is not universally valid. Whether GFA and ferromagnetism exhibit synergistic or competing behavior is ultimately governed by the local atomic configuration and its influence on nearest-neighbor Fe–Fe exchange interactions. Thus, the simultaneous achievement of high GFA and strong ferromagnetism is physically feasible, and the key lies in designing short-range ordered structures that favor both amorphous stability and strong exchange coupling through appropriate compositional design.

M_s_, H_c_, and ρ constitute another typical set of interdependent properties. The precipitation of α-Fe-like nanocrystals generally favors an increase in magnetization and a reduction in H_c_; however, the ρ of the crystalline phase is lower than that of the amorphous matrix. As the crystalline volume fraction increases, neighboring nanocrystals may gradually become interconnected and form highly conductive pathways, thereby compromising the high ρ required for high-frequency applications. Zhang et al. [50] identified an electrical percolation threshold of approximately 14.8% in Fe_82.5_B_12_P_2_C_1_Cu_0.5_Co_2_ alloys. This value is lower than the typical range observed in classical systems containing randomly distributed spherical conductive particles, which was attributed to the in situ nanocrystallization and continuous morphological evolution of this alloy system. When the crystalline volume fraction is below 14.8%, the intergranular amorphous layers maintain electrical isolation between the conductive crystalline phases. Once the threshold is exceeded, neighboring small crystalline nuclei aggregate and grow, gradually forming long-range continuous conductive pathways. Near this threshold, the average grain size is approximately 9.2 nm; M_s_ increases with fluctuations, H_c_ first decreases and then increases, whereas ρ decreases continuously (Figure 4c). Above the electrical percolation threshold, the nanocrystals progressively interconnect to form conductive pathways (Figure 4d), resulting in a rapid decrease in ρ accompanied by an increase in H_c_. Therefore, an optimal nanocrystallization window also exists, requiring a microstructural balance between magnetic softening and the retention of high electrical ρ.

## 3. Composition–Property Regulation and Mechanisms

### 3.1. Typical Compositions of High-B_s_ Nanocrystalline Alloys

The core design objective of high-B_s_ nanocrystalline alloys is not simply to increase the Fe content, but rather to establish a new balance among a high content of ferromagnetic elements such as Fe and Co, the GFA of the amorphous precursor, controllable crystallization, and uniform refinement of nanocrystals, thereby reducing the effective magnetic anisotropy and H_c_ [51,52]. To achieve this objective, the compositional design of high-B_s_ nanocrystalline alloys has evolved from the classical Cu–Nb strategy for synergistically controlling nucleation and grain growth toward more diverse approaches, including high-Fe-content design, engineering of high-temperature Fe–Co magnetic phases [53], and P–Cu synergistic tailoring of heterogeneous amorphous structures. These developments have continuously promoted the simultaneous optimization of magnetic performance and thermal stability in nanocrystalline alloy systems.

As one of the most representative nanocrystalline alloy systems, FINEMET was designed to address a key trade-off during the crystallization of FeSiB amorphous alloys: a high nucleation density is required to obtain a dense grain distribution, while grain growth must be effectively suppressed to prevent an increase in the effective magnetic anisotropy. To achieve this balance, Cu and Nb were simultaneously introduced into the FeSiB system, leading to the typical composition Fe_73.5_Si_13.5_B_9_Nb_3_Cu_1_ [54]. Cu promotes the formation of a high density of α-Fe(Si) nuclei, whereas Nb raises the crystallization temperature and suppresses grain growth. Their synergistic effect also inhibits the formation of Fe–metalloid compounds. As a result, a dual-phase structure consisting of randomly oriented α-Fe(Si) nanocrystals of approximately 10 nm and a residual amorphous matrix is obtained. This structure exhibits a B_s_ of approximately 1.24 T and an H_c_ as low as 0.53 A/m, together with high permeability and a low magnetostriction of approximately 2 × 10^−6^. The FINEMET system established the synergistic design concept in which different microalloying elements separately promote nucleation and inhibit grain growth, enabling deliberate control of nanocrystal size and providing a fundamental methodological framework for the subsequent development of nanocrystalline alloy systems.

Building on FINEMET, the most direct route to increasing B_s_ is to raise the Fe content while reducing the fractions of nonmagnetic elements such as Si, B, and Nb, leading to the development of NANOPERM alloys represented by Fe_91_Zr_7_B_2_ [55]. In this system, Zr and B help maintain the GFA of the amorphous precursor and restrict grain growth, while the total fraction of nonmagnetic elements is reduced to approximately 9 at.%. After crystallization, body-centered cubic (bcc) α-Fe-based nanocrystals with sizes of 10–20 nm are formed, B_s_ increases to 1.70 T, and H_c_ is 7.2 A/m at 1 kHz. Unlike FINEMET, which is designed to achieve extremely low H_c_, NANOPERM places greater emphasis on reducing magnetic dilution and increasing the Fe atomic fraction, at the expense of a certain increase in H_c_. This trade-off reflects the fundamental compromise between high magnetization and magnetic softening in high-B_s_ alloy design, and also indicates that simply increasing the Fe content is insufficient to achieve the lowest possible H_c_ simultaneously.

On the basis of NANOPERM, Co was introduced to partially replace Fe, resulting in the development of the (Fe,Co)-M-B-Cu-type HITPERM system, with Fe_44_Co_44_Zr_7_B_4_Cu_1_ as a typical composition [56]. The essence of this design is no longer simply to adjust elemental proportions, but to change the target magnetic phase from bcc α-Fe-based nanocrystals to ordered Fe–Co nanocrystals with greater magnetic stability at elevated temperatures. Its significance lies not in achieving the highest room-temperature B_s_, but in directly modifying the high-temperature magnetic properties of the nanocrystalline phase through substitution of the principal magnetic element. HITPERM represents an important advance in compositional design, extending the design strategy beyond grain-size control through alloy composition to the simultaneous tailoring of magnetic phase type and operating temperature range. This approach provides distinct advantages for specialized applications such as high-temperature power magnetic devices and aerospace power supplies.

For further enhancement of B_s_, large-atomic-size nonmagnetic elements such as Nb, Zr, and Mo become a bottleneck in compositional design because of their pronounced magnetic dilution effect. The NANOMET system was therefore developed to minimize the contents of these elements while retaining a stable amorphous precursor, giving rise to the FeSiBPCu system with typical compositions of Fe_83.3–84.3_Si_4_B_8_P_3–4_Cu_0.7_ [57]. Its key breakthrough lies in the synergistic addition of P and Cu, which promotes the formation of α-Fe clusters and compositionally enriched regions of approximately 2–3 nm in the as-quenched state, allowing nanocrystals of approximately 10–17 nm to form even without large amounts of grain-growth inhibitors. This strategy increases the Fe content to above 83 at.%, yielding B_s_ values of 1.88–1.94 T while maintaining H_c_ at approximately 7–10 A/m and a relatively low magnetostriction. The system also exhibits low core loss at 50 Hz, while the operating flux density corresponding to a rapid increase in core loss shifts from approximately 1.4 T to 1.7 T, demonstrating the advantage of a higher usable flux density enabled by the high B_s_. For distribution transformers, this high-B_s_ characteristic is beneficial for reducing the core cross-sectional area and material usage and can improve life-cycle energy efficiency by reducing core volume and material consumption.

### 3.2. Development Strategies for High-B_s_ Amorphous Alloys

Existing approaches to the design of high-B_s_ amorphous alloys can be broadly classified into two categories: traditional compositional design based on alloy physical and thermodynamic parameters, atomic size mismatch, and elemental interaction principles, and data-driven approaches developed in recent years, such as machine learning and high-throughput computation [58]. Traditional approaches adjust the contents of Fe and Co as well as the proportions of metalloids such as B, Si, C, and P to enhance B_s_ while maintaining sufficient GFA. Particular attention has been paid to the effects of increasing Fe content, optimizing the Fe/Co ratio, synergistically adjusting metalloid contents, and microalloying on B_s_, H_c_, and GFA [59].

**Figure 5:**
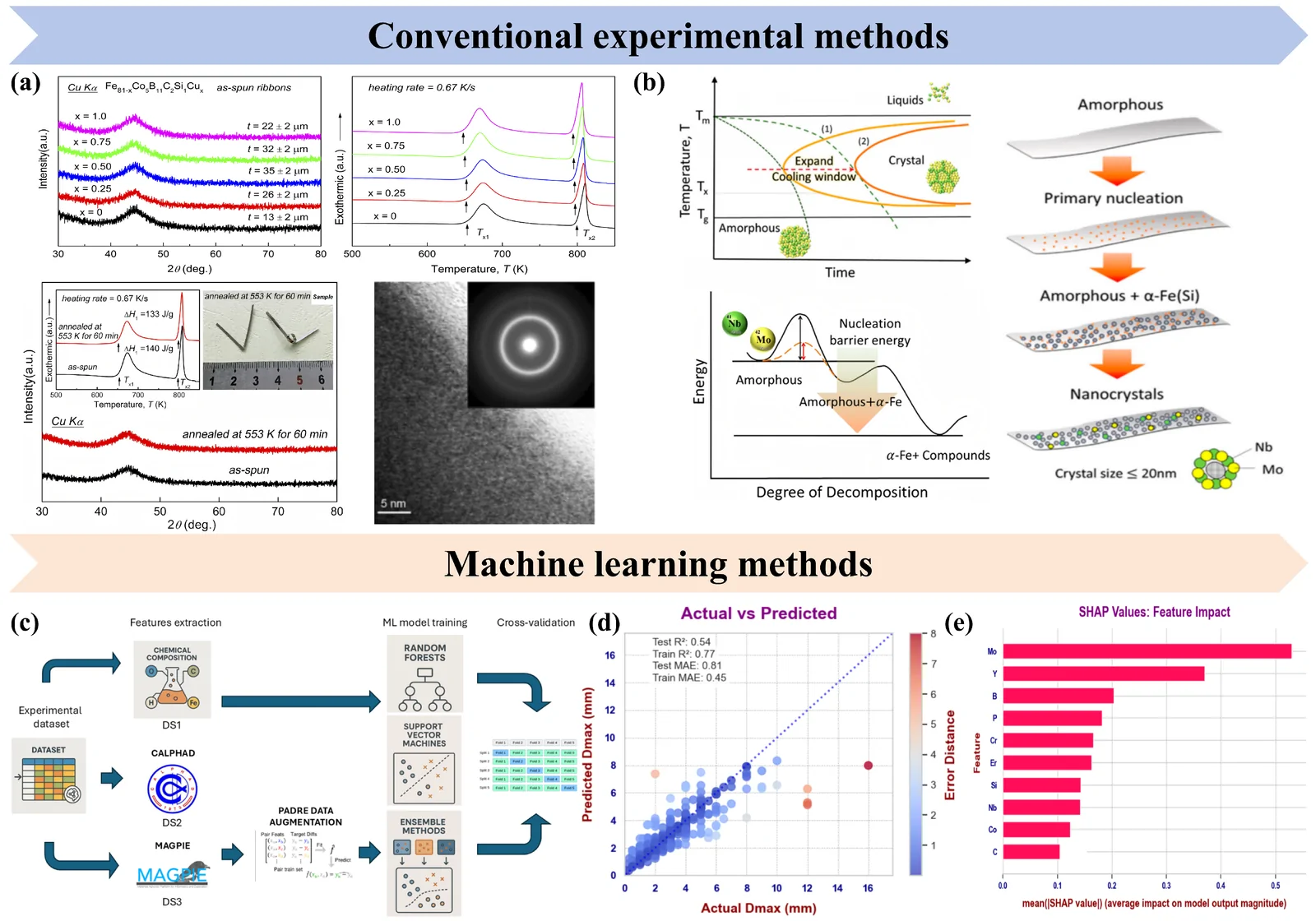
Design strategies for high-B_s_ Fe-based amorphous/nanocrystalline alloys. (**a**) Conventional experimental approaches used for the compositional design of FeCoBCSiCu amorphous alloys [60]. (**b**) GFA and crystallization behavior of FeSiBNbMoCu nanocrystalline alloys developed using conventional methods [62]. (**c**) Machine-learning workflow for the design and prediction of Fe-based amorphous alloys [67]. (**d**) Machine-learning predictions of Dmax for Fe-based amorphous alloys [67]. (**e**) Machine-learning-based ranking of compositional feature importance for Fe-based amorphous alloys [67].

The central challenge in traditional compositional optimization is to reconcile the trade-off between high B_s_ and low H_c_. The most common strategy is to vary the alloy composition and experimentally verify the resulting properties. A variety of conventional experimental approaches have been employed for this purpose, as illustrated in Figure 5a [60]. In addition, compositional design requires comprehensive consideration of the effects of individual elements on both GFA and magnetic softness. Li et al. [61] started from an Fe-B-based composition and partially replaced B with Si to balance GFA and magnetic softness, while optimizing the Fe/Co ratio to enhance exchange coupling. Cu was simultaneously introduced to provide nucleation sites, whereas V was added to suppress grain growth. After longitudinal magnetic-field annealing at 673 K for 10 min, a transitional structure was formed in which a small amount of bcc-Fe(Co,Si) nanocrystals, approximately 10–25 nm in size, were dispersed within the amorphous matrix. The crystalline volume fraction was only approximately 0.5%, while B_s_ reached 1.94 T and H_c_ decreased to 4.3 A/m. This compositional strategy does not rely on a high crystalline volume fraction to enhance B_s_. Instead, optimization of the Fe/Co ratio strengthens the local magnetic moments and exchange coupling, while the combined effects of Cu and V promote the formation of fine crystalline regions at a low volume fraction. This microstructure provides strong exchange coupling while maintaining low effective magnetic anisotropy and weak domain-wall pinning, thereby alleviating the trade-off between high B_s_ and low H_c_.

The microscopic mechanisms underlying microalloying highlight the importance of coordinated control over nucleation and grain growth. Related studies have shown that the addition of Mo, or the combined addition of Mo and Nb, enhances the stability of the amorphous phase by modifying the mixing enthalpy and atomic size mismatch. Meanwhile, Cu promotes cluster formation and provides heterogeneous nucleation sites for α-Fe(Si), whereas enrichment of Mo and Nb in the residual amorphous phase suppresses grain coarsening (Figure 5b). The resulting Fe_80_Si_4_B_12_Nb_1_Mo_2_Cu_1_ alloy forms nanocrystals with an average size of 18.16 nm after annealing, with M_s_ reaching 175.24 emu/g and H_c_ decreasing to 4.54 A/m [62]. These results indicate that the introduction of elements capable of stabilizing the residual amorphous phase and suppressing grain coarsening can help maintain a low effective magnetic anisotropy, thereby enabling a favorable balance between the optimized magnetic phase and the amorphous matrix in high-B_s_ amorphous alloys.

Introducing thermodynamic parameters into conventional compositional optimization can provide stronger physics-based guidance for composition screening. Zhang et al. [63] classified the constituent elements of Fe-based amorphous alloys into four categories and, using thermodynamic criteria, successfully narrowed the compositional window of Fe-P-C alloys to an Fe content of 78–80 at.% and a P content of 11–13 at.%, while achieving a critical casting diameter of 1.5 mm. This approach effectively reduces the candidate composition range for achieving high GFA. However, its primary focus remains the optimization of GFA, whereas further improvement in magnetic properties requires additional consideration of crystallization kinetics. Therefore, establishing an effective link between thermodynamic constraints and subsequent microstructural evolution remains a key issue for this type of design strategy.

In contrast to traditional empirical design, machine learning provides a new pathway for the development of high-B_s_ Fe-based amorphous/nanocrystalline alloys. A typical machine-learning workflow involves literature data collection, feature construction, and correlation analysis, followed by model training and prediction to rapidly map alloy composition to target properties. Guo et al. [64] developed an artificial neural network based on 885 alloy datasets and used it to screen 486,000 FeCoBSiCu composition combinations. The resulting Fe_68.2_Co_17.5_B_13_Si_0.5_Cu_0.8_ alloy developed a microstructure containing approximately 6.7% α-Fe(Co) after annealing, achieving a B_s_ of 1.96 T and an H_c_ of 1.2 A/m. Tang et al. [65] further incorporated processing parameters into an XGBoost model. Feature-importance analysis showed that Fe content and annealing temperature were the dominant factors governing B_s_, whereas annealing temperature and the amorphous/nanocrystalline structural state were most influential for H_c_. Based on these results, the designed Fe_85_Si_2_B_8.5_P_3.5_C_1_ alloy formed 9.9 nm α-Fe nanocrystals after annealing, with B_s_ = 1.82 T and H_c_ = 2.02 A/m. These findings indicate that machine learning has progressed from purely composition-based screening toward joint composition-process prediction, enabling a more direct correlation between structural evolution and magnetic-property responses.

At the stages of model selection and closed-loop optimization, comparisons among multiple algorithms showed that random forest generally outperformed k-nearest neighbors, decision trees, support vector machines, and linear regression in predicting saturation magnetization(M_s_), magnetostriction, H_c_, Curie temperature, grain size, and permeability [66]. This study employed 20-fold cross-validation and used R^2^ to evaluate the predictive performance of the different algorithms. However, even after two rounds of model updating and differential-evolution optimization, the random forest model still tended to underestimate H_c_ in the low-H_c_ region, indicating that sparse training data in this region continued to limit the model’s local predictive accuracy. In addition, Bobadilla et al. [67] developed a machine-learning workflow for Fe-based amorphous alloys using alloy composition, material parameters obtained from thermodynamic calculations, and basic elemental properties as model inputs, together with data augmentation, multiple machine-learning algorithms, and cross-validation for model training (Figure 5c). The study employed 10-fold cross-validation. The support vector regression model based on thermophysical features achieved a test R^2^ of 0.63 and an MAE of 0.72, whereas the best ensemble model further improved the performance to R^2^ = 0.69 and MAE = 0.69. The support vector regression model using alloy composition as input was also able to predict the critical casting size D_max_, thereby enabling rapid evaluation of alloy GFA, as shown in Figure 5d. Further SHAP analysis showed that Mo and Y made the most significant contributions to the prediction of D_max_, while B, P, and Cr also exhibited high feature importance (Figure 5e). Tang et al. [68] addressed the problem of data sparsity in the high-Fe-content region by using incremental learning to predict GFA and Gaussian process regression to predict B_s_, followed by iterative model updating through experimental feedback. The resulting Fe_85.5_Si_2_B_8.5_P_2_C_2_ alloy exhibited a measured B_s_ of 1.68 T, in close agreement with the predicted value of 1.67 T. These results demonstrate that machine learning can not only predict the GFA of _Fe_-based amorphous alloys across complex compositional spaces, but also identify key alloying elements that influence material properties, thereby providing a data-driven basis for candidate composition screening and the design of new alloys.

### 3.3. Regulatory Role of Metalloid Elements

In Fe-based amorphous/nanocrystalline soft magnetic alloys, metalloid elements such as Si, C, B, and P play important roles in determining the soft magnetic properties. Current research mainly focuses on the atomic-scale structural evolution associated with these elements and their effects on local atomic structure, nucleation behavior, and magnetic coupling [69]. Rather than affecting only a single property, metalloid elements can influence magnetic anisotropy and exchange coupling across multiple length scales by modifying the nearest-neighbor coordination environment of Fe, regulating short-range order in the amorphous matrix, and affecting the heterogeneous nucleation and growth of nanocrystals.

**Figure 6:**
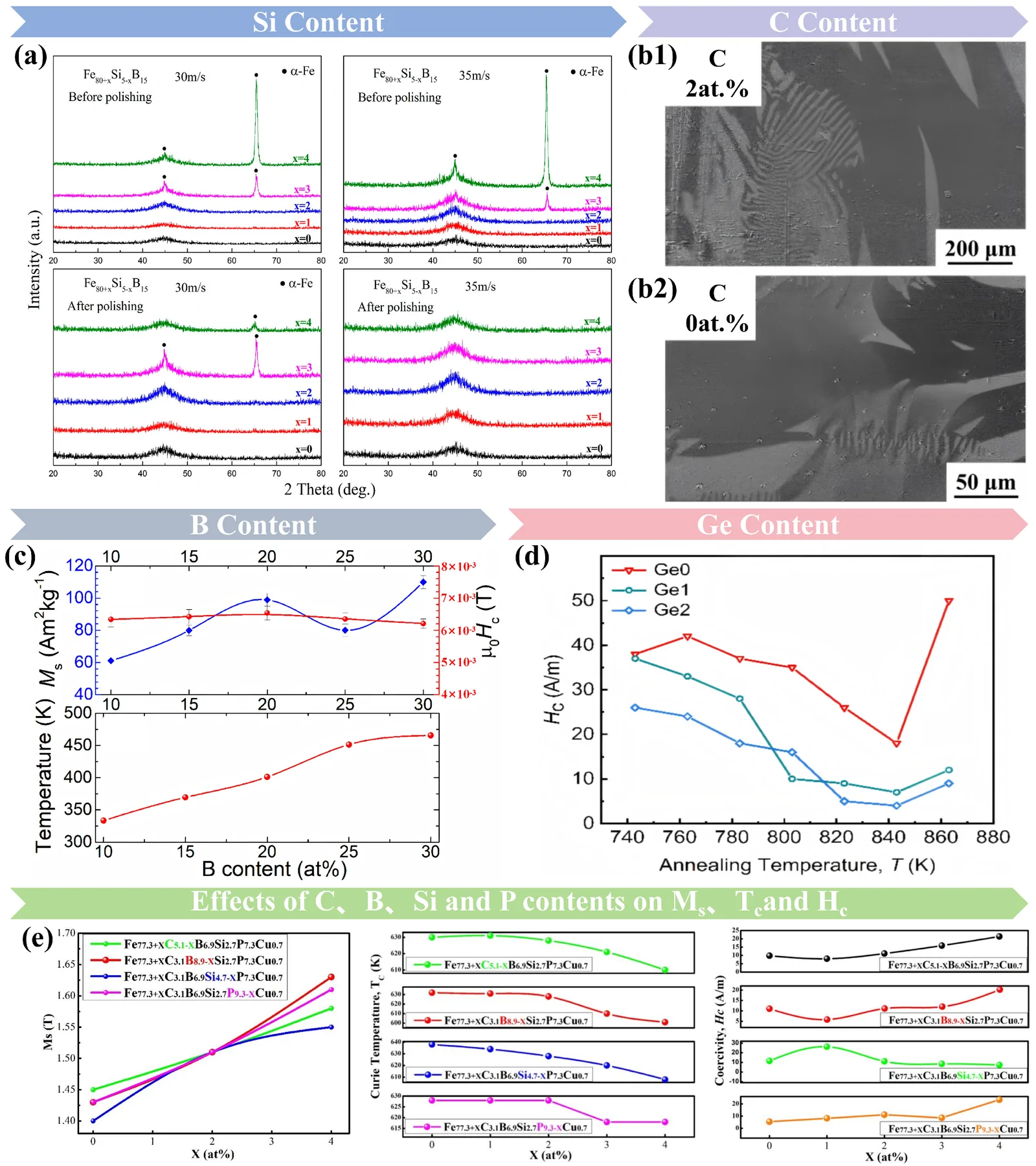
Effects of metalloid elements on the structure and soft magnetic properties of Fe-based amorphous/nanocrystalline alloys. (**a**) Effect of Si content on the phase constitution of as-quenched FeSiB alloy ribbons [42]. (**b1**,**b2**) Effect of C content on the magnetic domain structures of FeSiBPC amorphous ribbons [70]. (**c**) Effect of B content on M_s_ and H_c_ of FeZrNbB amorphous alloys [71]. (**d**) Effect of Ge content on H_c_ of FeSiBPNbC alloys [22]. (**e**) Effects of C, B, Si, and P contents on M_s_, T_c_, and H_c_ of FeCBSiPCu amorphous alloys [73].

Si and C exert pronounced effects on the local structure and magnetic properties of amorphous alloys. Jiang et al. [42] investigated Fe_80+x_S_i5−x_B_15_ (x = 0–4) alloys and found that decreasing the Si content while increasing the Fe content facilitated the precipitation of the α-Fe phase, indicating a gradual decrease in GFA (Figure 6a). At a ribbon-casting speed of 30 m/s, α-Fe could still be detected in the high-Fe, low-Si alloys even after surface polishing. When the wheel speed was increased to 35 m/s, however, all compositions exhibited pronounced amorphous diffuse-scattering features. These results indicate that an appropriate Si content helps suppress α-Fe precipitation and improve GFA, while a higher cooling rate further promotes the formation of the amorphous structure. Zhu et al. [70] further substituted C for Si in Fe_82_Si_6−x_B_9_P_3_C_x_ amorphous alloys and obtained fully amorphous structures at C contents of 2–6 at.%. At a C content of 2 at.%, the annealed alloy exhibited broader magnetic domains and straighter domain walls than the C-free alloy (Figure 6(b1,b2)), together with a B_s_ of 1.61 T and an H_c_ as low as 1.7 A/m. An appropriate C content can therefore improve GFA, promote stress relaxation, and reduce domain-wall pinning, thereby enhancing the soft magnetic properties.

Beyond single-element substitution, the effects of metalloid content on amorphous structure and soft magnetic properties are also reflected in changes in local ordering and interfacial segregation. In Fe_91−x_Zr_5_Nb_4_B_x_ amorphous alloys, increasing the B content from 10 to 20 at.% increased M_s_ from approximately 60 to 98 emu/g [71]. M_s_ then decreased to approximately 80 emu/g at 25 at.% B and increased again to 110 emu/g at 30 at.% B, as shown in Figure 6c. This nonmonotonic variation is associated with the precipitation of B2Zr secondary phases and the reconstruction of short-range order as the B content increases. The addition of Ge was also found to broaden the annealing temperature window of FeSiBPNbC alloys. At an optimal Ge content of 2 at.%, the alloy exhibited an H_c_ of 3 A/m and a B_s_ of 1.65 T, as shown in Figure 6d [22]. Determining the optimal concentration range of metalloid elements therefore essentially requires a balance between local structural modification and interfacial evolution. This understanding provides a theoretical basis for the compositional design of amorphous alloys with enhanced magnetic performance.

Beyond the effects of individual elements, the combined incorporation of multiple metalloids, including C, P, B, and Si, gives rise to more pronounced compositional coupling effects. In Fe_83.3_B_8_(C_x_P_y_Si_z_)_8_Cu_0.7_ amorphous alloys, variations in the relative proportions of these elements significantly affect the crystalline phase constitution and soft magnetic properties [72]. During nanocrystallization, the complex diffusion behavior arising from the coexistence of C, P, and Si collectively suppresses interface migration and grain growth. An appropriate compositional ratio is also beneficial for increasing Second crystallization temperature (T_x2_). The best overall performance was obtained for C1P6Si1, for which the annealed alloy exhibited a grain size of approximately 5.2 nm, a Bs of 1.70 T, and an H_c_ of 3 A/m. As shown in Figure 6e, in FeCBSiPCu amorphous alloys, the coordinated adjustment of C, B, Si, and P contents results in an overall increase in M_s_, whereas curie temperature (T_c_) generally decreases. H_c_ varies markedly with the type and relative proportion of the metalloid elements, indicating that their effects on magnetization, thermal stability, and magnetic softening are not synchronized [73]. The synergistic effect of multiple metalloid elements is therefore not a simple superposition of individual elemental effects, but rather arises from systematic coupling among their structural and magnetic roles.

First-principles molecular dynamics simulations provide further insight into the physical origin of this multi-element synergy. Simulations of FePB amorphous alloys [74] show that, as B is gradually replaced by P, the spatial distributions of Fe, B, and P atoms change. Although the amorphous structure retains long-range disorder, the local atomic arrangement undergoes progressive rearrangement. Because P has a larger atomic size and different chemical affinity, its introduction alters the coordination environment between Fe and the metalloid elements and promotes the formation of local short-range ordered structures. Adjusting the relative proportions of multiple elements can therefore further influence subsequent crystallization behavior and magnetic properties by reconstructing the local atomic configuration and chemical short-range order of the amorphous alloy.

### 3.4. Regulatory Role of Transition-Metal Elements

Transition-metal elements such as Co, Cu, Mo, and Nb act through mechanisms distinct from those of metalloid elements. Their roles include tuning ferromagnetic exchange coupling, promoting the heterogeneous nucleation of nanocrystals, and improving the thermal stability of the amorphous phase. By modifying grain size, grain-size distribution, and the state of the intergranular amorphous layers, these elements further influence random anisotropy and domain-wall pinning. In transformer core applications, appropriate additions of transition-metal elements can reduce H_c_ while maintaining a relatively high B_s_, thereby helping to reduce hysteresis and eddy-current losses and improve energy-conversion efficiency.

**Figure 7:**
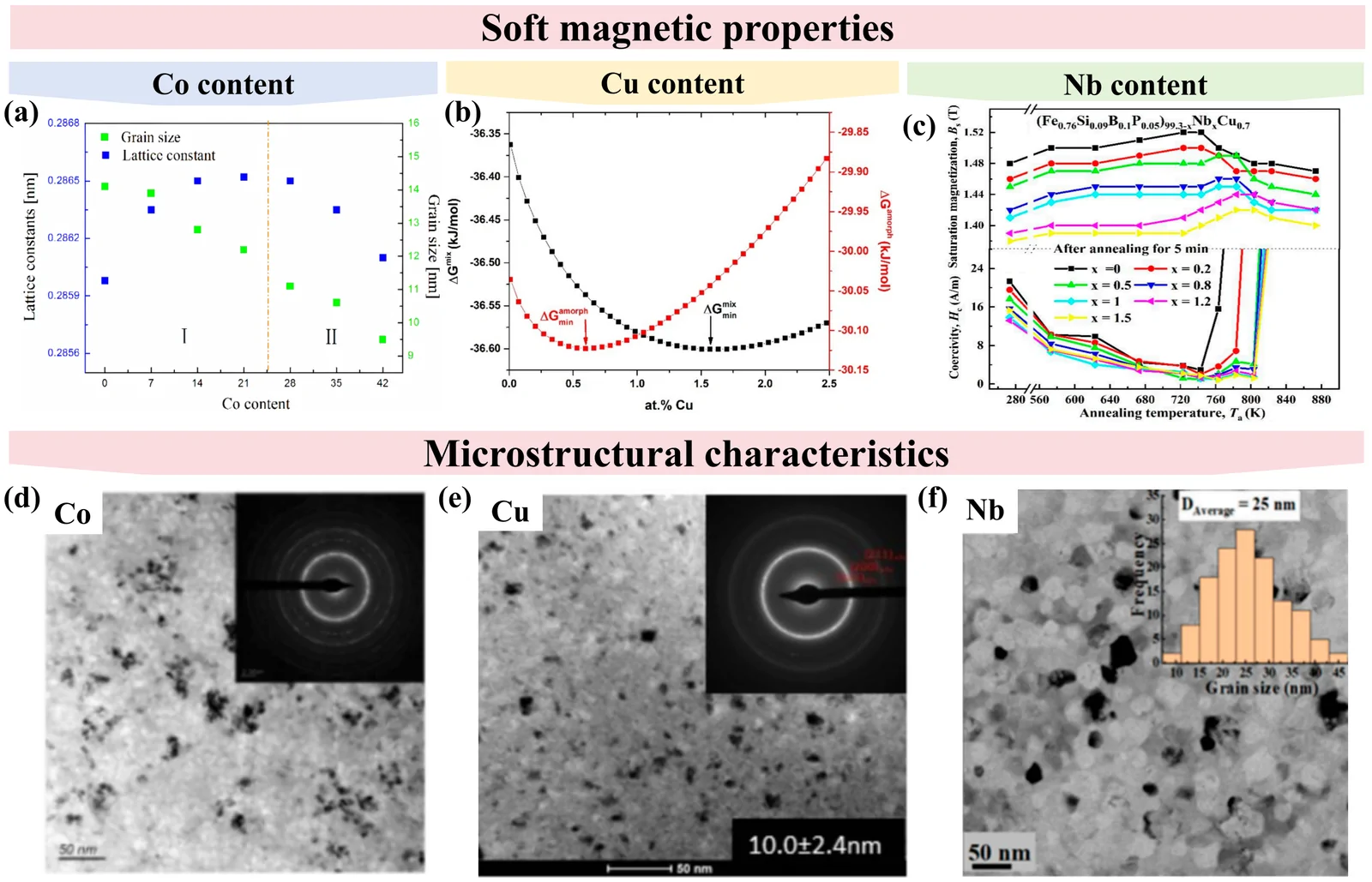
Effects of transition-metal elements on the structure and soft magnetic properties of Fe-based amorphous/nanocrystalline alloys. (**a**) Effect of Co content on the lattice constant and grain size of FeCoZrB alloys [76]. (**b**) Effect of Cu content on the free energies of FeNiNbCuSiB amorphous alloys [78]. (**c**) Effect of Nb content on B_s_ and H_c_ of FeSiBPNbCu alloys annealed at different temperatures [80]. (**d**) Effect of Co addition on the microstructure of annealed FeCoZrB alloys [76]. (**e**) Effect of Cu content on the microstructure of annealed FeNiNbCuSiB alloys [78]. (**f**) Effect of Nb content on the morphology and grain-size distribution of α-Fe(Si) nanocrystals in annealed FeSiBPNbCu alloys [80].

As an important ferromagnetic alloying element that partially substitutes for Fe in Fe-based amorphous alloys, Co can influence structural stability and soft magnetic properties by modifying the atomic structure and magnetic exchange interactions. Taking Fe_83.2−x_Co_x_B_10_C_6_Cu_0.8_ amorphous alloys as an example [75], H_c_ first decreases and then increases with increasing Co content, reaching a minimum of 12.9 A/m at 6 at.% Co, where a relatively uniform magnetic domain structure is also observed. With a further increase in Co content, the magnetic domains become distorted and domain-wall motion is hindered, causing H_c_ to increase to 19.13 A/m at 10 at.% Co. In Fe_84−x_Co_x_Zr_7_B_9_ alloys [76], the α-Fe(Co) grain size continuously decreases with increasing Co content, while the lattice constant changes from an increasing to a decreasing trend at approximately 21 at.% Co (Figure 7a), indicating that the Co content directly affects the crystallized structure. Excessive Co reduces the magnetic moments, while structural inhomogeneity, residual stress, and local anisotropy collectively enhance domain-wall pinning. Therefore, optimization of the Co content requires a balance between enhanced exchange interactions and structural uniformity.

Unlike Co, which mainly affects ferromagnetic exchange coupling, Cu influences structural evolution and magnetic properties primarily by controlling the nucleation of α-Fe. Shi et al. [77] systematically summarized the effects of Cu content on the overall properties and identified distinct performance regimes at different Cu concentrations. At low Cu contents (≤0.5 at.%), an insufficient number of Cu clusters limits α-Fe nucleation, resulting in higher H_c_ and requiring a higher annealing rate. In the intermediate range of 0.5–1.25 at.%, an appropriate Cu content produces sufficient Cu-enriched regions, promoting uniform α-Fe nucleation and grain refinement and thereby achieving relatively high B_s_ and low H_c_. In contrast, excessive Cu (≥1.5 at.%) promotes the formation of quenched-in nuclei, reduces GFA, and increases structural inhomogeneity. In Fe_72−x_Ni_8_Nb_4_Cu_x_Si_2_B_14_ alloys [78], the Gibbs free energy for amorphous formation and the Gibbs free energy of mixing reach minimum values at approximately 0.6 and 1.6 at.% Cu, respectively (Figure 7b), indicating that the Cu content significantly alters the thermodynamic conditions for amorphous formation and subsequent crystallization. These results indicate that optimization of the Cu content requires a balance between promoting nucleation and maintaining the stability of the amorphous precursor, which together determine the degree of grain refinement and the final soft magnetic properties.

In contrast, Nb microalloying modifies the nanocrystalline/amorphous dual-phase structure by changing the crystalline volume fraction of α-Fe(Si) and the thickness of the intergranular amorphous layers. In Fe_76.3−x_Si_9_B_10_P_4_Cu_0.7_Nb_x_ amorphous alloys [79], as the Nb content increases from 0 to 2.0 at.%, the crystalline volume fraction of α-Fe(Si) continuously increases from 34.6% to 57.8%, while the thickness of the intergranular amorphous layers decreases markedly from 14.9 nm to 2.8 nm. The increase in crystalline volume fraction indicates more extensive precipitation of α-Fe(Si), whereas the continuous thinning of the intergranular amorphous layers suggests a reduced degree of amorphous isolation between neighboring nanocrystals. Nb addition can therefore simultaneously adjust the degree of crystallization and the intergranular amorphous-layer thickness, thereby optimizing the nanocrystalline/amorphous dual-phase structure. As shown in Figure 7c, in the (Fe_0.76_Si_0.09_B_0.1_P_0.05_)_99.3−x_Nb_x_Cu_0.7_ system [80], the Nb content significantly affects B_s_ and H_c_ at different annealing temperatures. The best overall soft magnetic properties are obtained within the annealing-temperature range of 723–783 K, whereas H_c_ increases rapidly when the annealing temperature becomes excessively high.

The differences in the roles of these transition-metal elements can be further verified at the microstructural level. In their study of Fe_83_B_10−x_C_3_Si_3_P_1_Cu_x_ amorphous alloys, Shi et al. [77] found that the alloy containing 1 at.% Cu remained fully amorphous in the as-quenched state, whereas the alloy containing 1.5 at.% Cu already contained α-Fe nuclei with a size of approximately 4.1 nm. Chen et al. [81] investigated Fe_83.3_(Si_4_B_8_P_4_)_16−x_Cu_0.7_Mo_x_ amorphous ribbons after annealing and found that Fe_3_(B,P) phases formed in the Mo-free sample, whereas the sample containing 1 at.% Mo retained a distinct intergranular amorphous matrix, with the grain size decreasing from 22.7 to 21.2 nm. As shown in Figure 7d–f, Co addition reduces the size of α-Fe(Co) nanocrystals in FeCoZrB alloys and promotes their more uniform dispersion within the residual amorphous matrix. With increasing Cu content, the α-Fe nanocrystals in FNiNbCuSiB alloys remain fine and uniformly distributed, while the grain size decreases further. In FeSiBPNbCu alloys containing 0.5 at.% Nb, increasing the annealing temperature from 783 to 823 K causes the α-Fe(Si) grains to grow from approximately 19 to 25 nm, accompanied by a pronounced reduction in the residual amorphous phase. Overall, although different transition-metal elements act through different mechanisms, their contents must all be maintained within an appropriate range that balances their beneficial effects against compositional dilution. This represents a common principle in transition-metal microalloying design.

In summary, alloying elements regulate the structural evolution and soft-magnetic properties of Fe-based amorphous/nanocrystalline alloys through their effects on GFA, crystallization, nanostructure, and magnetic interactions. Their contents must be carefully optimized, as excessive additions may cause magnetic dilution, secondary-phase formation, or structural inhomogeneity. Table 1 summarizes their primary roles, representative beneficial contents/ranges, and adverse effects. These values are indicative rather than universal and depend on the specific base alloy system and processing conditions.

## 4. Heat-Treatment Regulation of Amorphous/Nanocrystalline Structure and Properties

The excellent soft magnetic properties of amorphous/nanocrystalline alloys are strongly influenced by heat treatment. For nanocrystalline alloys, heat treatment further determines the final magnetic properties by controlling grain size, crystalline volume fraction, and interfacial structure. H_c_ is particularly sensitive to residual stress, free volume, and nanocrystal size. Annealing therefore serves not only to relieve internal stress, but also to regulate atomic-scale ordering, induce nanocrystal precipitation, optimize magnetic domain structures, and reduce domain-wall pinning. In this way, heat treatment provides a critical link between the intrinsic magnetic potential of the material and its engineering performance.

### 4.1. Conventional Isothermal Annealing

Conventional isothermal annealing is the most fundamental and widely used heat-treatment method for inducing nanocrystallization in industrial production [82]. The underlying process is straightforward but critical: the amorphous precursor is heated to a target temperature near or above T_x_ but below T_x2_, held isothermally for a prescribed period, and transformed into the desired nanocrystalline microstructure through controlled crystallization kinetics. The key lies in the coordinated selection of annealing temperature and holding time, which jointly determine two critical microstructural parameters: grain size and crystalline volume fraction.

**Figure 8:**
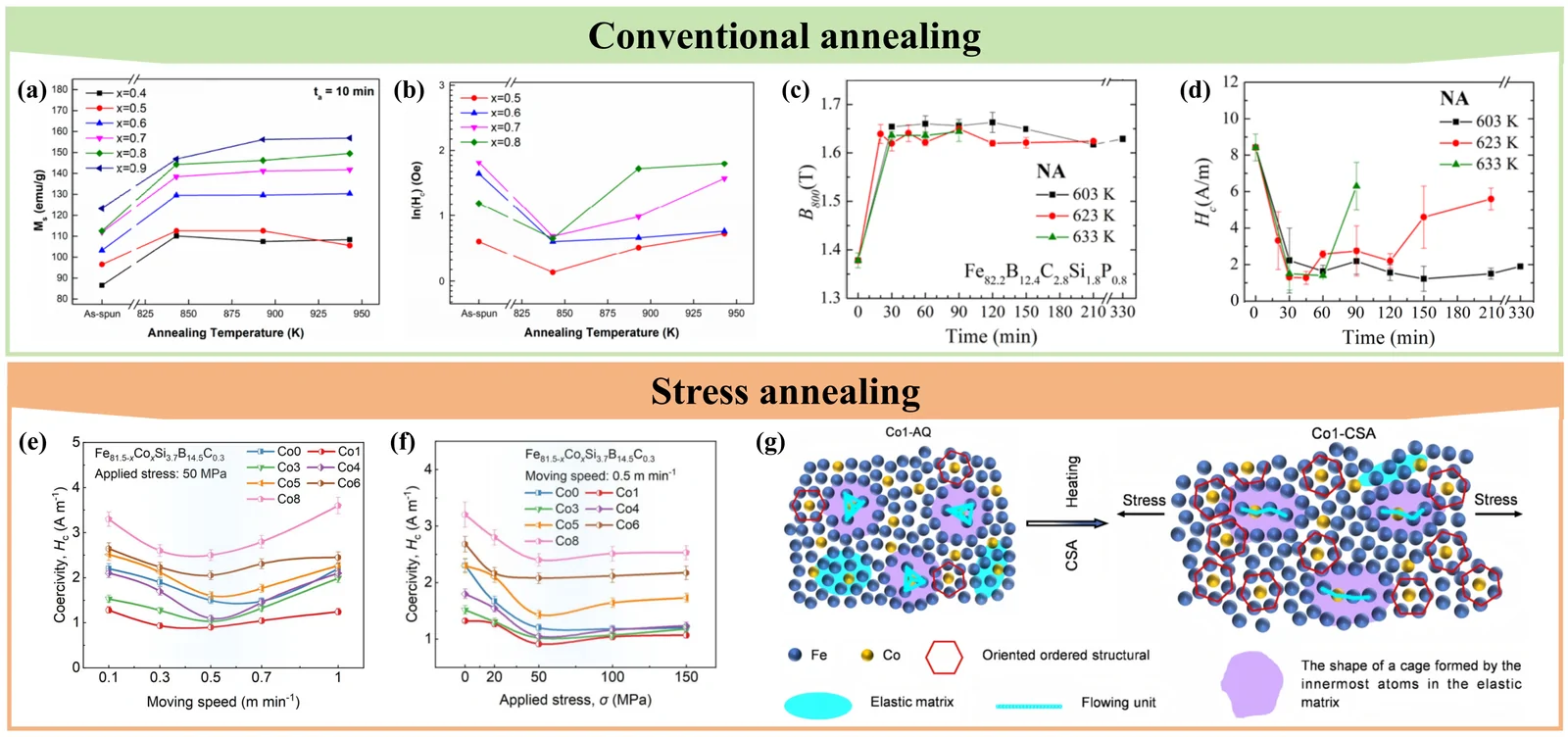
Tailoring the soft magnetic properties of Fe-based amorphous/nanocrystalline alloys through conventional and stress annealing. (**a**,**b**) Effects of conventional annealing temperature on M_s_ and H_c_ of FeCoBSiCP amorphous alloys [83]. (**c**,**d**) Effects of holding time during conventional annealing on B800 and H_c_ of FeBCSiP amorphous alloys [84]. (**e**,**f**) Effects of ribbon speed and applied tensile stress during continuous stress annealing on H_c_ of FeCoSiBC amorphous alloys [87]. (**g**) Schematic illustration of stress-annealing-induced atomic rearrangement and magnetic anisotropy formation [87].

Annealing temperature primarily determines the precipitated phase constitution and the resulting evolution of magnetic properties. The optimal temperature window arises from the kinetic competition between beneficial phase precipitation and detrimental phase transformations. Studies on (Fe_x_Co_1−x_)_72_B_19.2_Si_4.8_Nb_4_ alloys [83] showed that annealing at 893 K promotes the precipitation of the α-(Fe,Co) phase, increasing M_s_ from 112.5 emu/g in the as-quenched state to 156.8 emu/g (Figure 8a). However, when the annealing temperature is increased to 943 K, the precipitation of detrimental phases such as Fe_3_B and (Fe,Co)_23_B_6_ causes H_c_ to increase again (Figure 8b). This nonmonotonic behavior indicates that excessively low annealing temperatures provide insufficient structural relaxation and therefore limited property improvement, whereas excessively high temperatures promote detrimental phase precipitation and grain coarsening, resulting in property degradation. Conventional annealing therefore has a relatively narrow optimal temperature window, whose boundaries are governed by the competition between the kinetics of beneficial phase precipitation and those of detrimental phase formation.

The effect of annealing time is likewise governed by crystallization kinetics. Within the optimal annealing-temperature window, the isothermal crystallization process can be broadly divided into three stages. The initial stage is dominated by high-density nucleation, during which the grain number density increases rapidly. The intermediate stage is characterized by grain growth, with both grain size and crystalline volume fraction increasing toward their target values. In the final stage, nucleation is essentially saturated and grain growth gradually enters a slow coarsening regime dominated by Ostwald ripening. Studies on Fe_82.2_B_12.4_C_2.8_Si_1.8_P_0.8_ alloys [84] showed that B800 rapidly increases from approximately 1.37 T in the as-quenched state to 1.63–1.65 T after annealing and then remains essentially within the range of 1.62–1.66 T during subsequent holding (Figure 8c). At 603 K, H_c_ changes only slightly with holding time and remains as low as 1.9 A/m after 330 min. At 623 K, H_c_ decreases to 1.3 A/m after 30 min and remains below 3 A/m over a holding time of 30–120 min. When the temperature is increased to 633 K, however, H_c_ reaches 6.3 A/m after 90 min and subsequently increases rapidly (Figure 8d). These results indicate that conventional annealing requires an appropriate limitation of holding time while ensuring sufficient structural relaxation, so as to avoid excessive crystallization during prolonged heat treatment.

The deterioration of soft magnetic properties caused by over-annealing is an important engineering issue and originates from microstructural degradation induced by excessive temperature or prolonged holding. Hiramoto et al. [85] employed multiple characterization techniques, including TEM, APT, and SRXD, to investigate the microstructure of annealed Fe_84.8_Si_0.5_B_9.4_P_3.5_Cu_0.8_C_1.0_ alloys and revealed an intermittent nucleation behavior of α-Fe during isothermal annealing at 633–733 K. Notably, although the Cu clusters formed at higher temperatures are larger, they provide more effective nucleation sites for α-Fe and consequently result in finer grains. In contrast, smaller Cu clusters formed at lower temperatures are accompanied by coarser α-Fe grains. These observations indicate that Cu clusters larger than 3 nm formed at relatively high isothermal temperatures are more favorable for the rapid nucleation of a high density of fine grains, highlighting the advantage of high-temperature, short-time annealing for grain refinement and H_c_ reduction. Based on these microstructural evolution characteristics, isothermal annealing should employ an appropriate annealing temperature combined with a short holding time, provided that sufficient crystallization is achieved, in order to minimize grain size and effectively suppress boride precipitation.

Conventional annealing improves soft magnetic properties primarily through structural relaxation and controlled nanocrystallization. Studies of structural relaxation in Fe_76_Si_9_B_10_P_5_ alloys [21] showed that different annealing conditions produce amorphous states with different degrees of structural relaxation. At relatively low temperatures, insufficient atomic activation results in the coexistence of Fe-enriched and Fe-depleted regions, leading to nonuniform ferromagnetic coupling. Under appropriate annealing conditions at 748 K, the short- and medium-range structures undergo coordinated rearrangement, producing an exchange-coupled state with a more uniform distribution of strongly and weakly ferromagnetic regions and thereby effectively reducing magnetic anisotropy and H_c_. These results indicate that the improvement in soft magnetic properties during conventional annealing arises from the combined effects of nanocrystal precipitation and atomic ordering within the amorphous matrix. Process design must therefore balance structural relaxation and crystallization kinetics rather than relying solely on the degree of grain refinement.

### 4.2. Stress Annealing

Stress annealing is a heat-treatment process in which an external tensile or compressive stress is applied during annealing to induce magnetic anisotropy through coupled thermal and mechanical effects. In addition to promoting structural relaxation, stress annealing enables active control of magnetic anisotropy, which distinguishes it from conventional annealing.

Stress annealing promotes directional atomic rearrangement under an applied stress at temperatures above the creep-onset temperature of the amorphous alloy. Upon cooling, the resulting structural anisotropy is retained, enabling effective control of the magnetic domain structure. Studies on Fe_83−x_Co_x_B_10_Si_3_C_3_P_1_ alloys [86] showed that, although conventional annealing improves magnetic softness in low-Co alloys, it increases domain branching and domain-wall pinning in high-Co alloys. In contrast, stress annealing at 633 K for 20 min maintains H_c_ within 1.8–2.2 A/m and core loss within 0.09–0.11 W/kg at 1.0 T and 50 Hz, while effective permeability (μ_e_) is higher than that in both the as-quenched and conventionally annealed states. The curved or fingerprint-like magnetic domains observed in the as-quenched state originate from the coupling between magnetostriction and residual stress. Stress annealing reduces free volume, promotes the regular alignment of stripe domains along the ribbon axis, and generates a longitudinal easy magnetization axis through magnetoelastic coupling. These effects suppress local anisotropy fluctuations and domain-wall pinning, resulting in stable improvements in soft magnetic properties over a relatively wide compositional range.

Continuous stress annealing further extends the practical applicability of this approach. Taking Fe_80.5_Co_1_Si_3.7_B_14.5_C_0.3_ amorphous ribbons as an example [87], under a tensile stress of 50 MPa, H_c_ first decreases and then increases as the ribbon speed increases from 0.1 to 1.0 m/min, reaching a minimum at 0.5 m/min (Figure 8e). At this optimal speed, H_c_ also exhibits a nonmonotonic dependence on tensile stress as the stress increases from 20 to 150 MPa, reaching a minimum of 0.92 A/m at 50 MPa (Figure 8f). If the ribbon speed is excessively high, the effective heating time becomes insufficient, resulting in inadequate free-volume relaxation and residual-stress release. Conversely, an excessively low ribbon speed prolongs thermal exposure and promotes local enrichment of metalloid elements, thereby degrading the soft magnetic properties. Therefore, an appropriate combination of effective annealing time and applied tensile stress is essential for promoting structural relaxation and magnetic-domain rearrangement while minimizing H_c_.

The physical origin of this nonmonotonic dependence on stress level and thermal exposure time lies in the microstructural evolution driven by thermomechanical coupling. Applied tensile stress promotes the transformation of the magnetic domain structure from a randomly disordered as-quenched state toward a more uniformly ordered three-dimensional magnetization configuration. In combination with an appropriate content of ferromagnetic elements such as Co, it can also promote medium-range atomic ordering and further enhance stress-induced anisotropy (Figure 8g). The anisotropy induced by stress annealing is closely related to λ_s_ and the applied stress; however, a near-zero macroscopic λ_s_ does not necessarily imply that stress annealing is ineffective. Constrained elastic strain, stress-assisted atomic rearrangement, and local-phase magnetostriction can still generate induced anisotropy. Taking (Fe_1−x_Co_x_)_86_B_13_Cu_1_ nanocrystalline alloys as an example, as the Co content increases from 0 to 5 at.%, the overall λ_s_ increases from approximately +13 ± 2 ppm to +20 ± 2 ppm. Stress annealing can generate an induced anisotropy energy of up to approximately 460 ± 20 J/m^3^, indicating that the applied stress should be optimized in conjunction with the magnetostriction and structural-relaxation characteristics of the alloy. For alloys with relatively high λ_s_, the objective is not to maximize stress-induced anisotropy, but rather to reduce the effective λ_s_ under service conditions through compositional design and nanocrystallization while applying a relatively low annealing stress, thereby balancing effective anisotropy control with long-term stability under stress.

Stress annealing provides effective control of magnetic properties at the laboratory scale, but its industrial scale-up still faces several key challenges. During continuous annealing of wide ribbons, maintaining uniform transverse tension is difficult, and even small fluctuations can cause the local magnetic anisotropy to deviate from the target state. Transverse temperature gradients can also produce nonuniform thermal histories and different degrees of structural relaxation across the ribbon width, resulting in macroscopic variations in magnetic properties. In practical wound cores, bending and thermal stresses introduced during annealing and service can interact with the externally applied tensile stress. In addition, the induced anisotropy may gradually relax during prolonged exposure to elevated temperatures, potentially compromising long-term reliability. Practical scale-up may therefore benefit from online tension monitoring, transverse temperature mapping, and controlled cooling to minimize stress and thermal nonuniformity. Validation on wound cores under representative processing and service conditions is also important for assessing the transferability and stability of the induced anisotropy at the component level. Achieving coordinated control of tensile stress, annealing temperature, line speed, and cooling rate while accommodating complex core geometries remains essential for translating stress-annealing technology from laboratory studies to engineering applications.

### 4.3. Magnetic Field Annealing

Magnetic field annealing is an external-field-assisted heat-treatment process in which a directional magnetic field is applied during structural relaxation. Through the interaction between the applied magnetic field and atomic magnetic moments, magnetic field annealing promotes local structural rearrangement and the preferential alignment of magnetic moments along the field direction, thereby inducing uniaxial magnetic anisotropy. Under thermally activated conditions, the applied field modifies the local atomic arrangement and magnetic-moment orientation, allowing the magnetic domains to develop a preferred orientation during annealing.

**Figure 9:**
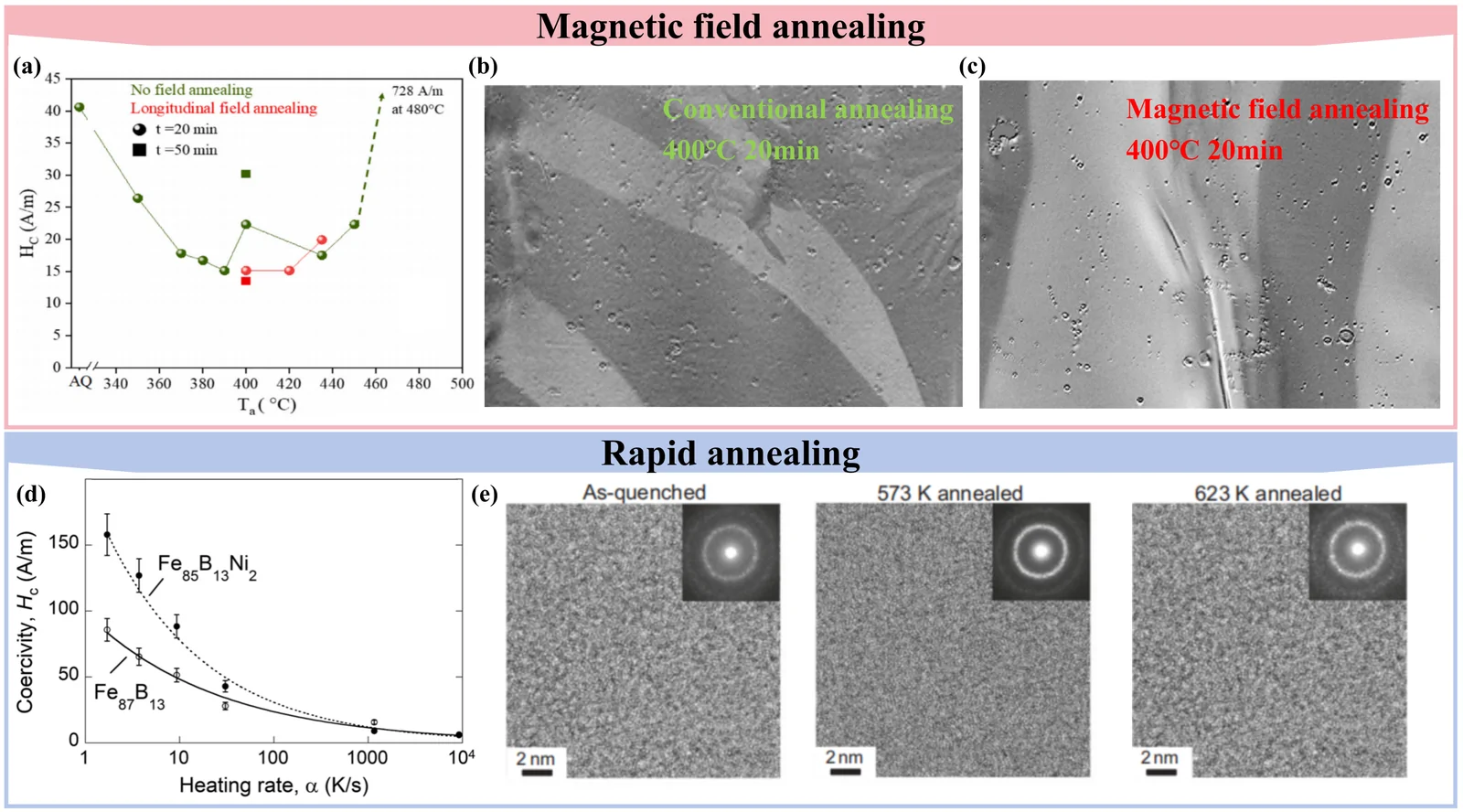
Tailoring the soft magnetic properties of Fe-based amorphous/nanocrystalline alloys through magnetic field annealing and rapid annealing. (**a**) Effect of zero-field and magnetic-field annealing on H_c_ of FeBPSiC amorphous alloys [4]. (**b**,**c**) Comparison of the magnetic domain structures of FeBPSiC amorphous alloys after conventional and magnetic-field annealing [4]. (**d**) Effect of heating rate during rapid annealing on H_c_ of FeB-based amorphous alloys [92]. (**e**) Effect of rapid-annealing temperature on the microstructure of FeSiPBCuC amorphous alloys [93].

The applied magnetic field strength determines the extent of the response, and even extremely weak fields can produce pronounced effects. Studies on Fe_80_Si_9_B_11_ amorphous ribbons annealed at 653 K for 1 h under a longitudinal magnetic field of 0–1 T [88] showed that, although conventional annealing already significantly increased B_1600_ and permeability (μ) while reducing H_c_, the application of an extremely weak magnetic field led to further improvement. However, the additional changes became progressively smaller as the field strength increased toward 1 T. In samples annealed under a magnetic field of 2.5 × 10^−4^ T, spherically symmetric ordered clusters were formed, with localized regions exhibiting a certain degree of structural ordering, which may be associated with improved local structural uniformity. Saha et al. [4] investigated Fe_80_B_12_P_4_Si_2.7_C_1.3_ amorphous ribbons and found that, after conventional annealing at 400 °C for 20 min, H_c_ was approximately 22 A/m, whereas the application of a 16 kA/m longitudinal magnetic field reduced H_c_ to approximately 15 A/m (Figure 9a). Meanwhile, relatively narrow and nonuniform magnetic domains were still observed after conventional annealing, whereas magnetic field annealing produced significantly wider domains, fewer domain walls, and weaker domain-wall pinning (Figure 9b,c). These results indicate that an applied magnetic field can further improve the soft magnetic properties of Fe-based amorphous alloys by reorganizing magnetic anisotropy and promoting a more regular magnetic domain configuration.

T_c_ limits the effective temperature range of magnetic field annealing, while the primary role of the applied magnetic field is to reduce magnetic anisotropy rather than directly enhance B_s_. After Fe_83.2−x_Co_x_B_10_C_6_Cu_0.8_ amorphous ribbons were annealed for 10 min under a 0.1 T longitudinal magnetic field [89], the applied field had little influence on B_s_, indicating that its main effect was not to change M_s_ itself. After magnetic field annealing at 360 °C, the Co-free alloy and the alloy containing 6 at.% Co exhibited B_s_ values of 1.75 T and 1.86 T, respectively, while their H_c_ values decreased to 3.4 A/m and 2.2 A/m, respectively. For the latter alloy, H_c_ was reduced by approximately 84% relative to the as-quenched state. These results demonstrate that magnetic field annealing promotes magnetic softening primarily by reducing magnetic anisotropy rather than by directly increasing B_s_. Process design should therefore focus on controlling magnetic anisotropy rather than maximizing magnetization.

The effect of magnetic field annealing on the H_c_ of Fe-based amorphous alloys is not governed by a single factor, but arises from the combined effects and competition among structural relaxation, surface effects, internal stress, field-induced magnetic anisotropy, and cluster evolution. During the initial stage of annealing, free volume decreases, residual stress inherited from the as-quenched state is released, domain-wall pinning is weakened, and H_c_ decreases. As the temperature increases, the field-induced uniaxial anisotropy becomes stronger, promoting the preferential alignment of magnetic moments along a specific direction and facilitating a more uniform magnetization process. However, when the temperature becomes excessively high or the induced anisotropy is too strong, local structural inhomogeneity and magnetic anisotropy contributions may increase again, causing H_c_ to rise. Therefore, magnetic field annealing has an optimal processing window, within which the magnetic-softening effect of structural relaxation must be balanced against the orientational effect of field-induced anisotropy to achieve effective control of H_c_.

### 4.4. Rapid Annealing

Although conventional isothermal annealing offers the advantage of process simplicity, its inherently narrow processing window for high-B_s_ nanocrystalline alloys, typically only 20–30 K, poses considerable challenges for batch-process control [90]. The development of rapid annealing provides an alternative approach based on kinetic control to address this limitation.

Rapid annealing exploits high heating rates to control the competition between nucleation and grain growth. A large degree of thermal superheating during rapid annealing can markedly increase the homogeneous nucleation rate, while the short holding time limits atomic diffusion and grain growth. The combined effect promotes the formation of a high density of fine nanocrystals. However, as the annealing time is prolonged, grain coarsening and enhanced domain-wall pinning cause H_c_ to increase again. Studies on Fe_84_Si_3_B_10_P_2_Cu_1_ and Fe_84.5_Si_3_B_9.5_P_2_Cu_1_ precursor ribbons [91] showed that, for ribbons with a thickness of 30 μm and a heating rate of approximately 100 °C/s, the Fe84.5 alloy maintained a B_s_ of approximately 1.90 T and a relatively low H_c_ after rapid annealing at 480–520 °C for 30 s, indicating a relatively broad temperature window. After rapid annealing at 480 °C for 30 s, Fe84Si3B10P2Cu1 exhibited an α-Fe crystalline volume fraction of approximately 61% and an average grain size of 23.1 ± 4.8 nm, with B_s_ = 1.88 T and H_c_ = 8.6 A/m. Taking Fe_87_B_13_ and Fe_85_B_13_Ni_2_ amorphous alloys as further examples [92], increasing the heating rate from 1.7 K/s to above 10^3^ K/s reduced the average grain size from 30–40 nm to approximately 20 nm, while H_c_ decreased from 80–160 A/m to below 8 A/m (Figure 9d). These results directly demonstrate that increasing the heating rate can promote magnetic softening by suppressing grain growth. They also indicate that pre-existing α-Fe nuclei in the precursor do not necessarily lead to abnormal grain growth during subsequent annealing. The intense crystallization competition induced by rapid heating can effectively restrict grain coarsening, allowing partially crystallized precursors to develop a uniform and fine nanocrystalline microstructure.

The rapid-annealing temperature also governs the structural evolution from the amorphous to the nanocrystalline state. When Fe_84.7_Si_2.8_P_3.8_B_7.8_Cu_0.7_C_0.2_ amorphous ribbons are rapidly heated at approximately 120 K/s and held for 3 min [93], the amorphous structure is largely retained after annealing at 573–653 K, whereas a large number of α-Fe(Si) grains precipitate and grow markedly when the temperature is increased to 723 K (Figure 9e). These observations indicate that rapid annealing also has an optimal temperature window, while excessively high temperatures promote excessive crystallization and grain coarsening.

The kinetic advantage of rapid annealing arises from the strong sensitivity of the nucleation rate to thermal superheating and the diffusion-controlled nature of grain growth. A high heating rate combined with a short holding time enhances nucleation while restricting grain growth, allowing high-Fe-content alloys that lack diffusion-inhibiting elements such as Nb to retain a fine-grained microstructure. This reflects the distinct role of rapid annealing in high-B_s_ alloy systems. Using Cu- and Nb-free Fe_50_Co_33_B_17_ amorphous alloy as a model system, this kinetic advantage was further demonstrated [94]. When the heating rate exceeded 300 K/s and the alloy was annealed at 813 K for 3 s, B_s_ reached 1.87 T and H_c_ decreased to 5.6 A/m. The resulting nanocrystals had an average size of approximately 8.0 ± 3.6 nm and consisted of randomly oriented α-Fe(Co) grains that were densely and uniformly distributed. In contrast, conventional annealing produced coarser and more sparsely distributed grains, with H_c_ generally exceeding 30 A/m. However, such a heating rate is not universally applicable to all alloy compositions. The underlying reason is that greater thermal superheating increases both the thermodynamic driving force for the amorphous-to-crystalline transformation and the homogeneous nucleation rate, whereas the short holding time restricts subsequent grain growth. Cu provides heterogeneous nucleation sites, while Nb suppresses grain growth. Therefore, variations in Cu and Nb contents alter the heating rate and effective processing window required to achieve a fine nanocrystalline structure. These comparisons demonstrate that the combination of enhanced homogeneous nucleation under large thermal superheating and restricted grain growth during short-time annealing can produce fine nanocrystalline structures even without Cu and Nb additions, providing an important basis for the heat-treatment design of low-cost, high-B_s_ alloy systems.

Nevertheless, the engineering implementation of rapid annealing still faces substantial challenges. In terms of heating uniformity, maintaining a uniform temperature distribution across wide ribbons or large cores is difficult at high heating rates, and local overheating or underheating can lead to nonuniform grain-size distributions and variations in magnetic properties. In terms of equipment capability, although second-scale annealing enables high processing throughput, the integration of continuous feeding, heating, and cooling remains insufficiently mature. Process robustness is another major concern. Although some alloy compositions exhibit a relatively broad annealing-temperature range, the effective time–temperature window for rapid or flash annealing is generally narrow, placing much greater demands on process-control precision. At present, induction heating, laser scanning, and direct-current heating each offer distinct advantages and limitations, and rapid-annealing technology as a whole remains in the transition from laboratory-scale studies to pilot-scale validation.

Overall, the four heat-treatment methods exhibit distinct advantages and limitations, as summarized in Table 2. Conventional annealing is industrially mature, stress and magnetic-field annealing enable anisotropy control, and rapid annealing promotes grain refinement with shorter processing times. The listed temperature/time conditions are indicative rather than fixed and should be selected relative to the alloy-specific T_x_, considering composition, ribbon thickness, and required magnetic anisotropy.

## 5. Amorphous/Nanocrystalline Composite Structures

### 5.1. Effects of Amorphous/Nanocrystalline Structures on Soft Magnetic Properties

The amorphous/nanocrystalline composite structure is a fundamental microstructural feature of nanocrystalline alloys. Even at a high degree of crystallization, residual amorphous interfacial layers with a thickness of approximately 1–2 nm can remain between adjacent grains, indicating that nanocrystalline alloys essentially possess an amorphous/nanocrystalline dual-phase structure [95]. Precise control of the crystalline volume fraction and intergranular spacing can effectively modify the exchange-coupling length and magnetization process, thereby improving the soft magnetic properties [96].

**Figure 10:**
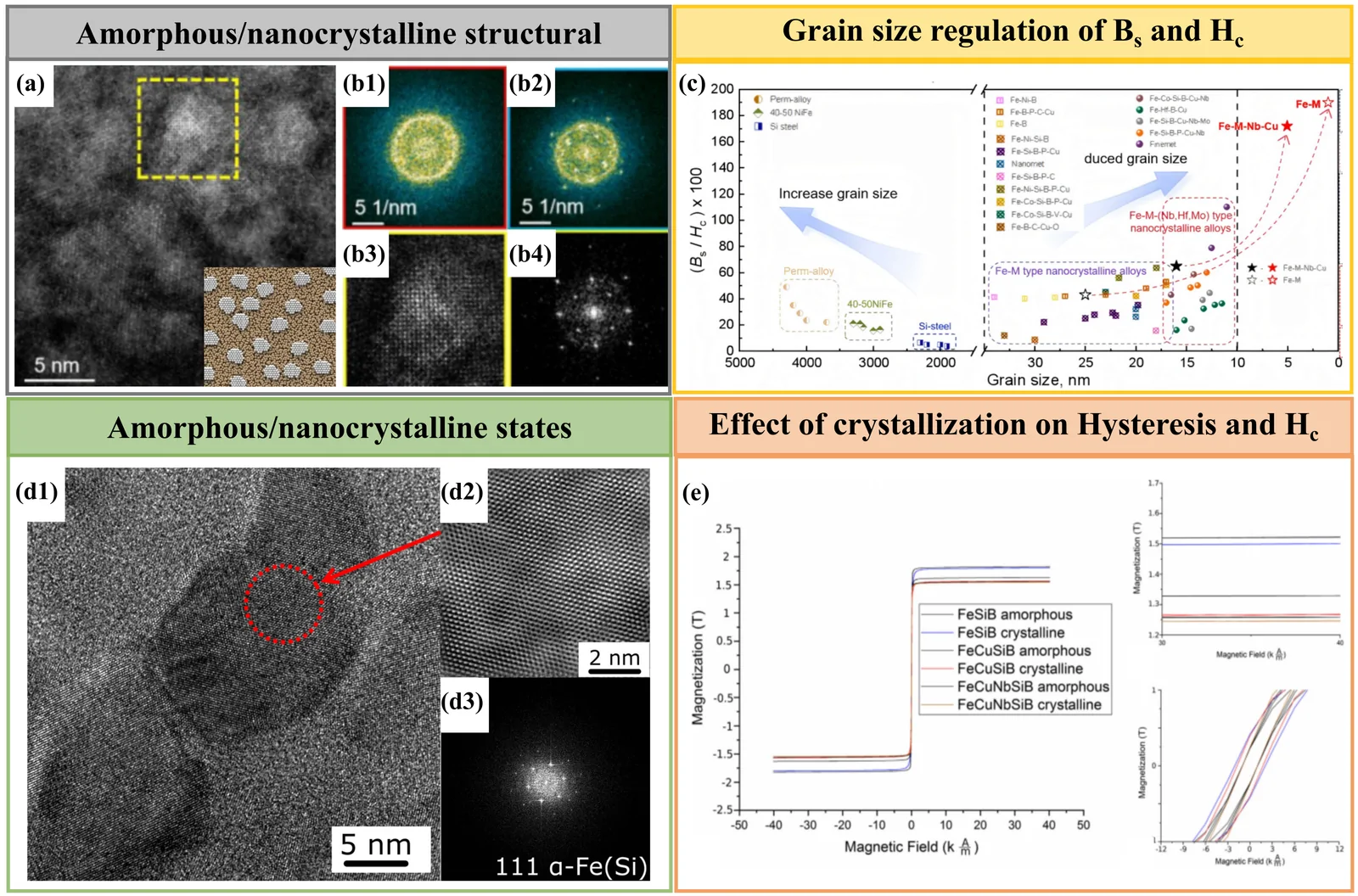
Effects of amorphous/nanocrystalline structures on the soft magnetic properties of Fe-based soft magnetic alloys. (**a**) Microstructure of the amorphous/nanocrystalline composite matrix, with the inset showing a schematic illustration of the structure, the yellow dashed box indicating the region selected for further structural analysis [47]. (**b1**–**b4**) Fourier transform and inverse Fourier transform characterization of the crystalline region/amorphous matrix composite structure [47]. (**c**) Relationship between grain size and the comprehensive performance parameter B_s_/H_c_ in ultrafine nanocrystalline structures [47]. (**d1**–**d3**) High-resolution characterization of nanocrystals embedded in the amorphous matrix of FeCuNbSiB amorphous/nanocrystalline alloys [97]. (**e**) Effects of different crystallization structures on the hysteresis loops and H_c_ of FeCuNbSiB amorphous/nanocrystalline alloys [97].

The amorphous/nanocrystalline composite structure is critical for achieving a favorable combination of high B_s_ and low H_c_ in Fe-based soft magnetic alloys. A single-phase amorphous structure lacks long-range order and magnetocrystalline anisotropy and therefore generally exhibits low H_c_ and high electrical ρ, both of which are beneficial for reducing magnetic losses. However, the dilution of Fe by metalloid elements limits B_s_. The precipitation of strongly ferromagnetic nanocrystalline phases can increase B_s_, whereas coarse grains, nonuniform grain distributions, or the formation of magnetically hard phases enhance magnetocrystalline anisotropy and domain-wall pinning, leading to an increase in H_c_. An ideal microstructure therefore consists of fine nanocrystals uniformly dispersed within an amorphous matrix. After rotational magnetic-field annealing, Fe_78.65_Co_4_Si_2_B_13_Cu_1.35_Nb_1_ forms a composite structure in which nanocrystals are uniformly embedded in a continuous amorphous matrix (Figure 10a), and Fourier-transform analysis confirms that the bright regions correspond to body-centered cubic nanocrystals (Figure 10(b1–b4)) [47]. The high density and uniform dispersion of ultrafine nanocrystals preserve the contribution of the ferromagnetic phase while reducing local magnetic anisotropy, providing a structural basis for combining high B_s_ with low H_c_. This composite structure affects not only static magnetic properties but also frequency-dependent dynamic losses. The highly resistive amorphous interfacial layers can interrupt conductive pathways between nanocrystals, thereby increasing the overall ρ of the alloy and suppressing classical eddy-current losses at high frequencies. Therefore, coordinated control of the amorphous interfacial-layer thickness and crystalline volume fraction is essential for balancing high-frequency loss and magnetic performance.

A major challenge in tailoring the amorphous/nanocrystalline composite structure is the simultaneous control of crystalline volume fraction and grain size, which generally evolve together during annealing and are therefore difficult to adjust independently. Analysis of the relationship between grain size and soft magnetic properties shows that, when the grain size is below the exchange-correlation length of approximately 35 nm, further grain refinement can significantly improve the figure of merit B_s_/H_c_ (Figure 10c). Ultrafine grains help maintain a strong contribution from the ferromagnetic phase while reducing the effective magnetocrystalline anisotropy through exchange averaging, thereby alleviating the trade-off between high B_s_ and low H_c_. However, this advantage depends on the coordinated control of grain size, crystalline volume fraction, and residual amorphous-phase fraction rather than on simply minimizing grain size. In frequency-dependent loss behavior, an increase in crystalline volume fraction is often accompanied by nanocrystal coarsening, which strengthens domain-wall pinning and increases hysteresis loss. Conversely, an excessively low crystalline volume fraction results in a larger fraction of amorphous matrix, which may increase magnetostriction and excess loss. An ideal microstructure for high-frequency applications should therefore retain a sufficiently high amorphous-phase fraction to maintain high ρ while incorporating uniformly distributed ultrafine nanocrystals to sustain high B_s_ and low H_c_, thereby enabling low loss over a broad frequency range. In (Fe_0.8_Co_0.2_)_85_B_12_Si_2_V_0.5_Cu_0.5_ alloys [61], nanocrystals with sizes of 10–25 nm and a volume fraction of only 0.5% are surrounded by numerous crystal-like ordered regions smaller than 5 nm. These structures collectively enhance local magnetic exchange interactions, resulting in B_s_ = 1.94 T and H_c_ = 4.3 A/m. This result indicates that a high B_s_ does not necessarily require a high crystalline volume fraction; a small fraction of crystalline units together with surrounding crystal-like ordered regions can also strengthen exchange interactions and reduce the effective anisotropy.

The continuous evolution of crystallization within such composite structures further determines the soft magnetic properties. After FeCuNbSiB amorphous alloy is annealed at 873 K [97], α-Fe(Si) nanocrystals several nanometers in size form within the amorphous matrix (Figure 10(d1–d3)), demonstrating that fine nanocrystals and the residual amorphous phase can coexist to form a stable amorphous/nanocrystalline composite structure. Further comparison of the hysteresis loops shows that, after relatively coarse crystallization in FeSiB and FeCuSiB alloys, H_c_ increases from approximately 1.5 kA/m to approximately 3 kA/m. In contrast, in the FeCuNbSiB alloy, H_c_ remains essentially unchanged before and after crystallization because the grain size remains below 20 nm (Figure 10e). This indicates that maintaining fine nanocrystals separated by the amorphous matrix can effectively suppress crystallization-induced magnetic hardening. When Fe_82–84_B_13–15_Si_1_C_1–2_Cu_1_ alloys form a nanocluster/amorphous structure with characteristic cluster sizes of approximately 4 nm after relaxation annealing [98], they can maintain B_s_ > 1.7 T and H_c_ < 7.8 A/m while exhibiting relatively high initial permeability (μ_i_). With further precipitation and coarsening of α-Fe, H_c_ increases and μ_i_ decreases rapidly. After multiphase compounds such as Fe_3_(B,C) and Fe_2_B form, the soft magnetic properties deteriorate markedly. This structural evolution also strongly affects frequency-dependent losses. As crystallization progresses, the amorphous interfacial layers gradually become thinner or even disappear, reconnecting conductive pathways and reducing electrical ρ, which leads to a sharp increase in high-frequency eddy-current loss. At the same time, enhanced domain-wall pinning associated with grain coarsening also increases high-frequency hysteresis loss. This mechanism is an important contributor to the deterioration of soft magnetic properties caused by excessive crystallization. During the structural evolution from a fully amorphous state to a nanocluster-containing state and finally to a more highly crystallized state, an optimal crystallization window exists for soft magnetic performance. Excessive crystallization increases magnetocrystalline anisotropy and domain-wall pinning through grain coarsening and multiphase precipitation.

The amorphous matrix provides a low-anisotropy background, nanocrystals contribute high magnetization, and ultrafine grains together with amorphous/crystalline interfaces reduce the effective anisotropy through exchange coupling. From the perspective of high-frequency applications, the high electrical ρ of the amorphous matrix and the high M_s_ of the nanocrystalline phase are complementary. Through precise control of crystalline volume fraction, amorphous interfacial-layer thickness, and grain size, both the static magnetic properties, including B_s_ and H_c_, and the frequency-dependent dynamic losses can be optimized. Structural design should therefore focus on stabilizing the amorphous matrix, producing uniformly distributed ultrafine nanocrystals, and suppressing magnetically hard phases, thereby combining low effective anisotropy with high magnetization to achieve high B_s_, low H_c_, high μ_e_, and low core loss.

### 5.2. Pre-Existing Nuclei/Ordered Structures

Traditional nanocrystallization processes generally regard the amorphous precursor as a homogeneous, disordered, and isotropic medium, with nucleation and grain growth during annealing considered to be primarily governed by processing parameters such as temperature, time, and heating rate. However, an increasing body of experimental evidence indicates that the microscopic state of the amorphous precursor, particularly the presence of pre-existing nuclei, medium-range order, or compositional fluctuations, can profoundly influence subsequent crystallization behavior.

**Figure 11:**
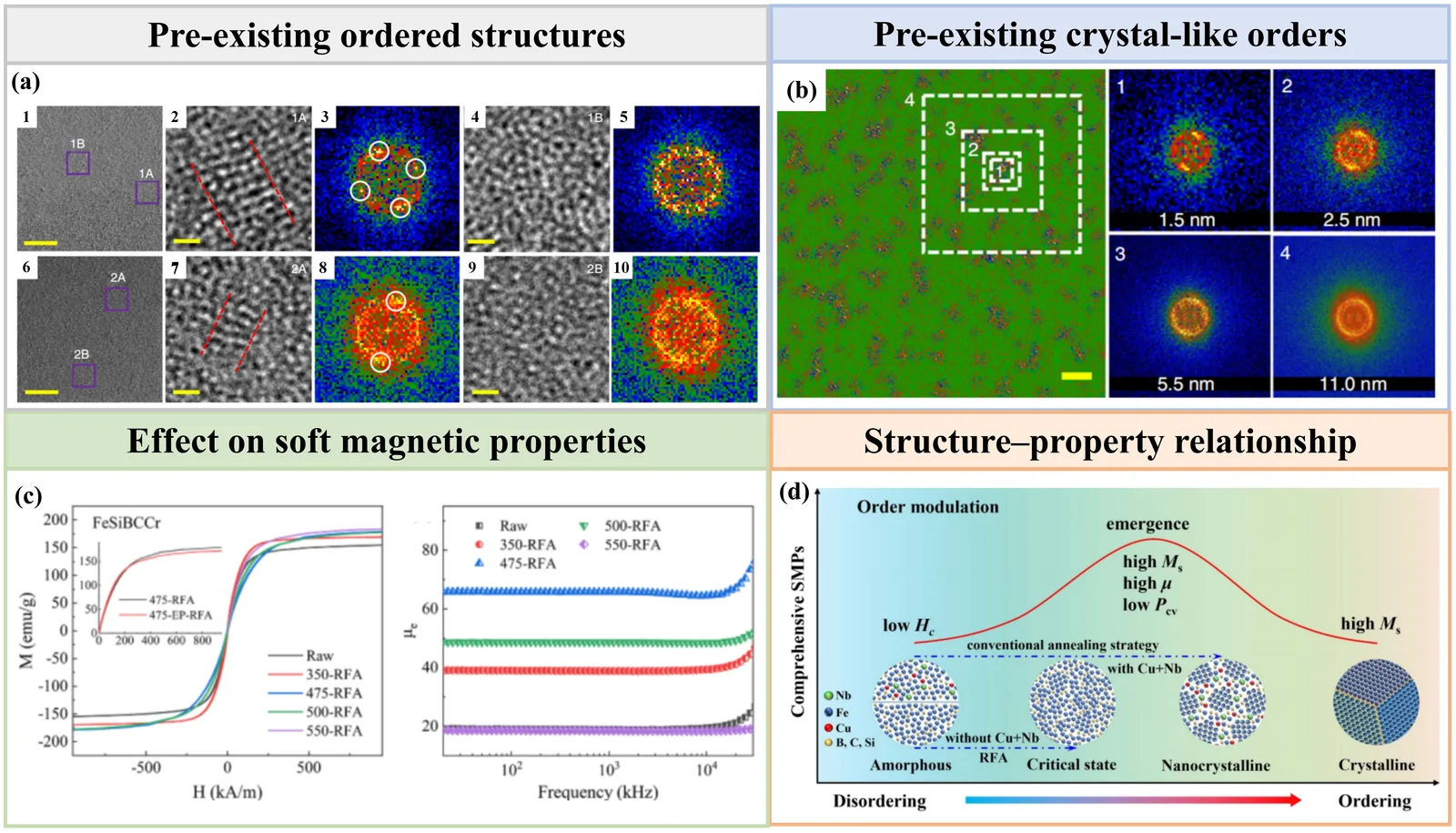
Effects of pre-existing nuclei/ordered structures on the nanocrystallization behavior and soft magnetic properties of Fe-based amorphous/nanocrystalline alloys. (**a**) HRTEM images and corresponding FFT patterns of pre-existing nanocrystals/locally ordered structures in FeNiPC amorphous alloys, with colored boxes marking selected regions, dashed lines indicating lattice fringes, and circles highlighting diffraction spots [100]. (**b**) Crystal-like ordered structures revealed by autocorrelation analysis and FFT patterns acquired from regions of different sizes in FeNiPC amorphous alloys [100]. (**c**) Magnetization curves and frequency dependence of μ_e_ for FeSiBCCr amorphous alloys under different rotating magnetic-field annealing conditions [102]. (**d**) Mechanism by which crystal-like ordered structures regulate the structural evolution among amorphous, critical, nanocrystalline, and crystalline states and optimize the comprehensive soft magnetic properties of M_s_, μ, H_c_, and core loss [102].

The concept of pre-existing nuclei has deepened the understanding of crystallization in amorphous alloys. During rapid solidification by planar flow casting, with cooling rates of approximately 10^5^–10^6^ K/s, small crystalline embryos may form during quenching when the alloy composition deviates from the optimal glass-forming range and become frozen within the amorphous matrix [99]. During subsequent annealing, these as-quenched nuclei can more readily overcome the critical nucleation barrier and enter the growth stage, thereby significantly altering the crystallization pathway. Similar locally ordered structures have been directly observed in Fe-based amorphous alloys. In Fe_50_Ni_30_P_13_C_7_ metallic glass [100], HRTEM revealed numerous locally ordered regions approximately 1–1.5 nm in size, which formed during casting and were uniformly dispersed within the amorphous matrix. As shown in Figure 11a, the ordered regions 1A and 2A exhibit clear lattice fringes, with discrete diffraction spots appearing in the corresponding FFT patterns, whereas the adjacent regions 1B and 2B retain typical disordered amorphous contrast and diffuse halos. These observations indicate that a material that appears macroscopically amorphous may still contain pre-existing nanoscale crystal-like ordered structures. Further autocorrelation analysis shows that these locally ordered structures exhibit pronounced scale dependence. As shown in Figure 11b, when the FFT analysis region is restricted to approximately 1.5–2.5 nm, diffraction features associated with local ordering are relatively pronounced. As the analysis scale is increased to 5.5–11 nm, the diffuse rings arising from the amorphous matrix gradually become dominant, and the signals from the nanoscale ordered structures are increasingly masked by spatial averaging. This demonstrates that a fully amorphous structure identified by conventional XRD or SAED performed over a relatively large area does not necessarily imply the absence of pre-existing nuclei or locally ordered structures.

A surface pre-crystallized layer represents a typical type of pre-existing structure, and its formation is closely related to melt-spinning conditions. When the wheel speed is reduced to 30 m/s, an α-Fe surface-crystallized layer containing 5–10 nm pre-existing crystals or clusters forms on the free surface. During subsequent annealing, a large number of these pre-existing crystals grow simultaneously and compete with one another within the surface layer, while the suppression of grain-boundary migration by Nb and Mo further limits grain coarsening. For a surface-crystallized sample containing 2 at.% Mo, H_c_ remains within 1.5–4 A/m after annealing at 560–580 °C for 10 min, while μ increases to 22,000–28,000. These results indicate that the effect of pre-existing crystals strongly depends on their size and spatial distribution. Fine, high-density pre-existing nuclei favor the development of a uniform microstructure through competitive growth, whereas coarse or sparsely distributed crystals are more likely to undergo abnormal grain growth.

Medium-range order in amorphous alloys, referring to ordered structures on the scale of approximately 1–3 nm, including icosahedral clusters and crystal-like configurations, lacks long-range translational symmetry but can nevertheless act as a precursor to crystallization and significantly influence nucleation behavior. Extending the concept from surface pre-crystallization to the deliberate construction of locally ordered structures, segregation of minor magnetic and interfacial elements can further regulate the formation and stability of nuclei. After transverse magnetic-field annealing at 853 K, Fe_75.5_Co_0.5_Mo_0.5_Cu_1_Nb_1.5_Si_13_B_8_ forms an α-Fe(Co)/amorphous dual-phase structure with an average grain size of 11.8 nm and a crystalline volume fraction of 71.2% [101]. Co is enriched within the nanocrystals, whereas Mo and Nb segregate near the interfaces, indicating that elemental partitioning around pre-existing ordered structures can further restrict grain growth and stabilize the fine-grained microstructure. Molecular dynamics simulations show that the average crystallization incubation time decreases from 2.6 to 1.8 ns under an applied magnetic field of 1 T. The resulting alloy achieves B_s_ = 1.42 T and H_c_ = 0.9 A/m. These results indicate that pre-existing ordered structures not only provide favorable nucleation sites, but that the coordinated partitioning of Co, Mo, and Nb around these structures also influences nucleus formation and subsequent grain growth, providing an important structural basis for obtaining fine and uniformly distributed nanocrystals.

For alloy systems lacking nucleation-promoting and diffusion-inhibiting elements such as Cu and Nb, adjusting the degree of order within the amorphous matrix itself provides another strategy for precursor design. As shown in Figure 11c [102], when FeSiBCCr amorphous alloy evolves from the initial amorphous state to a critically ordered state produced by rotating magnetic-field annealing at 475 °C, both its magnetization and μ_e_ improve. The increase in local ordering enhances ferromagnetic exchange interactions, while the amorphous matrix remains dominant and therefore avoids the introduction of strong magnetocrystalline anisotropy. The structural evolution from the amorphous state through the nanocrystalline state to the crystalline state, as shown in Figure 11d, indicates that a higher degree of crystalline order is not necessarily more favorable. Instead, an appropriate balance among the size, number density, and degree of order of the ordered regions is required. By maintaining the structure in a critical state between the fully amorphous and clearly crystallized states, low H_c_, high μ, high M_s_, and low Pcv can be achieved simultaneously, providing a new structural design strategy for optimizing the soft magnetic properties of Fe-based amorphous/nanocrystalline alloys.

Pre-existing crystals, atomic clusters, and locally ordered structures can significantly influence the subsequent crystallization behavior of Fe-based amorphous/nanocrystalline alloys. The conventional view is that surface crystallization or pre-existing crystals in as-quenched ribbons tend to introduce microstructural inhomogeneity and degrade soft magnetic properties. Recent studies, however, indicate that when these pre-existing crystals or ordered regions are sufficiently small, spatially well controlled, and restricted during annealing by slowly diffusing elements or interfacial segregation, they can participate in nucleation and grain refinement, thereby improving the nanocrystalline microstructure and soft magnetic properties.

### 5.3. Gradient and Multilayer Structures

While the amorphous/nanocrystalline composite structure and the regulation of pre-existing nuclei still pursue optimization within a homogeneous microstructural framework, gradient and multilayer structures represent a representative strategy for intentionally constructing spatial heterogeneity [103]. Although this strategy shows potential from the perspective of physical mechanisms, it currently remains primarily a conceptual design exploration and is fundamentally distinct from the homogeneous structural regulation that has already achieved industrial application. By establishing gradient distributions of microstructure, composition, or stress along the ribbon thickness or in-plane directions, and leveraging the coupling effects between spatial inhomogeneity and magnetoelasticity, magnetic property combinations that are difficult to achieve in homogeneous materials may potentially be achieved.

**Figure 12:**
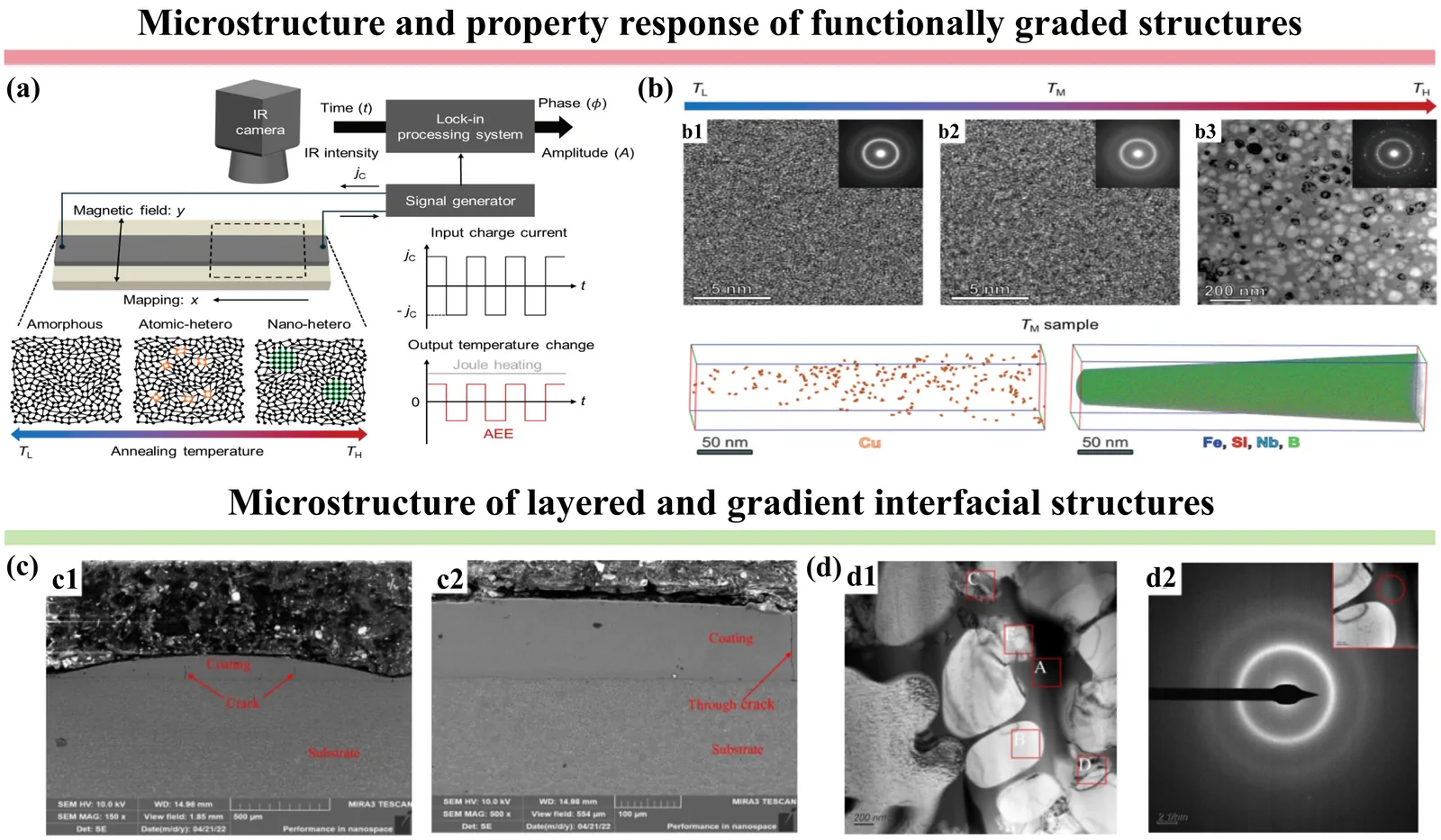
(**a**) Schematic illustration of the functionally graded structure formed in FeSiBNbCu amorphous ribbons by one-dimensional temperature-gradient annealing, showing a continuous evolution from the amorphous state through an atomic-scale heterogeneous state to a nanoscale heterogeneous state [105]. (**b**) BF-STEM images of FeSiBNbCu ribbons from different temperature regions and APT elemental distributions in the intermediate-temperature region, (**b1**–**b3**) BF-STEM images from the low-, intermediate-, and high-temperature regions, respectively. [105]. (**c**) Gradient layered structure of FeCoCrMoCBY amorphous alloy transitioning gradually from the substrate to the amorphous coating, (**c1**) Cross-sectional view showing the coating, crack, and substrate, (**c2**) enlarged view showing a through crack. [106]. (**d**) BF-TEM images and corresponding SAED patterns of the amorphous/crystalline heterointerfacial structure comprising the amorphous phase, bcc-Fe(Co,Cr), and other crystalline phases, (**d1**) BF-TEM image of the transition zone, (**d2**) SAED pattern of region A, In (**d1**), A denotes the black amorphous phase, B the white bcc-Fe(Co,Cr) phase, and C–E the gray crystalline phases. [106].

The compressive stress effect induced by surface crystallization is a representative engineering phenomenon that has been demonstrated experimentally in laboratory-scale gradient structures. During the annealing of amorphous ribbons or cores, the surface often preferentially crystallizes and undergoes volume contraction due to preferential oxidation, selective volatilization of elements, or temperature gradients, thereby forming biaxial compressive stress under the constraint of the internal amorphous matrix. In positive magnetostriction Fe-based amorphous alloys, this compressive stress induces in-plane magnetic anisotropy through magnetoelastic coupling, altering the magnetic domain configuration. Selective oxidation induced by air annealing at 280 °C in Fe_80_Co_3_Si_3_B_10_P_1_C_3_ amorphous cores forms a surface layer structure consisting of a crystallized oxide layer, an amorphous oxide layer, and an amorphous matrix [104]. O is mainly enriched at the surface, Fe diffuses outward to form Fe_2_O_3_ and Fe_3_O_4_, and a Co-rich, Fe-depleted region appears at the oxidation front. Moderate oxidation is beneficial for improving magnetic induction and permeability, while the oxide layer can reduce domain-wall pinning and eddy current losses. However, excessive oxidation thins the effective amorphous matrix and increases H_c_ to 15.4 A/m. Nevertheless, an optimal time window exists for air annealing, and its regulation logic requires additional consideration of the influence of oxidation degree on the surface layer structure and effective amorphous layer thickness. After temperature-gradient annealing, Fe_79_Si_4_B_14_Nb_2_Cu_1_ amorphous ribbons can form a gradient structure that continuously evolves from a fully amorphous state to an atomic-scale heterogeneous state and then to a nanoscale heterogeneous state along the spatial direction (Figure 12a) [105]. STEM and APT shown in Figure 12b further reveal that Cu nanoclusters appear in the intermediate region, while a coexisting structure of amorphous and Fe-based crystals forms in the high-temperature region. This continuous regulation may help mitigate abrupt structural and local stress changes, thereby potentially providing a structural basis for reducing domain-wall pinning and improving H_c_ and μ. However, these effects have so far been demonstrated only in laboratory-scale small samples, and spatial gradient control with equivalent precision has not yet been achieved in industrial continuous production.

Multilayer structures extend the concept of gradient regulation toward active structural design. By alternately depositing amorphous layers of different compositions during ribbon preparation, functionally graded composite materials may be constructed in the thickness direction. The crystallization behavior of each layer may be differentially regulated through compositional differences: high-B_s_ layers form high-B_s_ nanocrystals after annealing to provide high magnetization, while high-glass-forming-ability layers remain amorphous or partially crystallized to maintain high ρ and a stress-buffering function. Artificially constructed alternating amorphous/crystalline layers further regulate interfacial constraints and elemental interdiffusion. For example [106], as shown in Figure 12c, a gradient interface gradually transitioning from planar crystals, columnar crystals, and equiaxed crystals to an amorphous layer forms between the Fe_41_Co_7_Cr_15_Mo_14_C_15_B_6_Y_2_ amorphous alloy and the substrate. Its TEM and SAED further confirm that the transition zone is composed of an amorphous phase and crystalline phases such as bcc-Fe(Co,Cr) (Figure 12d). Such gradient/layered interfaces can reduce abrupt interfacial property changes and stress concentration, potentially suppressing magnetoelastic anisotropy and domain-wall pinning. Müller et al. [107] found that a critical thickness exists for alternating amorphous/crystalline layers. When the sublayer thickness decreases from 250 nm to 15 nm, the structure remains intact and hardness increases; below 15 nm, interfacial integrity is lost, and the synergistic effect degrades into a mixed effect. The interfacial blocking effect has been systematically verified in B-Fe-Ag multilayers, where the Ag layer thickness determines the interfacial blocking effect of the B-Fe-Ag multilayers [108]. When the Ag layer is less than 5 nm, it is discontinuous, and B can laterally diffuse into the Fe-B layer to alter the magnetization behavior. When it reaches 5 nm, a continuous blocking layer forms, and further thickening has little effect on magnetic properties. The structure between crystalline Ag layers consists of two layers: residual amorphous B and amorphous Fe-B. Interfacial layer continuity appears to be critical to effective diffusion blocking. When discontinuous, blocking fails; when continuous but excessively thick, magnetic properties are diluted due to the increased nonmagnetic volume. Although the interfacial effects in these thin-film systems possess clear physical implications, whether their conclusions can be directly transplanted to bulk ribbons and guide large-scale production still requires further engineering verification.

It is worth pointing out that the engineering application of gradient and multilayer structures is still in the exploratory stage. The thickness and stress state of the surface crystallized layer are extremely sensitive to annealing conditions, resulting in poor process controllability. The preparation of multilayer ribbons involves complex multi-channel planar flow casting equipment, and the manufacturing cost and efficiency are still far from industrialization. However, the spatial heterogeneity regulation design concept represented by gradient and multilayer structures offers an alternative to the conventional emphasis on microstructural homogenization in soft magnetic materials and may provide a useful design framework for future high-performance amorphous/nanocrystalline soft magnetic materials.

## 6. Performance Evaluation and Processing Challenges for Transformer Applications

The soft magnetic advantages of amorphous/nanocrystalline alloys must ultimately be realized and validated in transformer products [109]. From laboratory samples to industrial magnetic cores, the materials undergo multiple processing steps, including ribbon fabrication, core winding and forming, annealing treatment, and impregnation and curing, each of which may significantly affect the soft magnetic properties. Meanwhile, actual transformer operating conditions, including nonsinusoidal excitation, direct current (DC) bias, and environmental stresses, impose additional requirements on material performance beyond those under standard testing conditions.

### 6.1. Special Material Requirements Under Transformer Operating Conditions

Standard soft magnetic property tests are generally conducted under sinusoidal excitation, no-load conditions, and at room temperature, whereas actual transformer operating conditions differ significantly from these conditions [109]. A thorough understanding of these differences and their mechanisms of influence on material performance is essential for translating materials research into transformer applications.

Loss separation and evaluation at power frequencies and medium to high frequencies are primary concerns in transformer core design. At power frequencies (50/60 Hz), hysteresis loss generally accounts for 60–80% of the total loss, and reducing H_c_ is therefore the primary objective in optimizing distribution transformer cores. However, with the development of power electronic transformers and solid-state transformers, operating frequencies have extended to the kHz range and even tens of kHz. Eddy current loss increases rapidly, while excess loss rises sharply at high frequencies because of the nonuniformity of domain wall motion. Consequently, the importance of optimizing ρ and the magnetic domain structure increases significantly, whereas the priority of B_s_ decreases relatively. Studies of renewable energy converter operating conditions under complex electromagnetic excitation have revealed the evolution of losses under nonsinusoidal conditions. Tests on a 50 kVA amorphous alloy core at 50, 100, 150, and 200 Hz showed that core loss increased with increasing frequency and peak magnetic flux density. Hysteresis loss was the dominant component, eddy current loss remained relatively small at low frequencies but increased markedly with frequency, and excess loss was relatively low. When interharmonics were superimposed on the 50 Hz fundamental component, the peak magnetic flux density of the core increased and saturation occurred earlier, resulting in decreased μ and increased core loss. These results indicate that nonsinusoidal excitation alters the dynamic magnetization state of the core and aggravates local saturation, causing the actual loss to exceed that under sinusoidal excitation. For non-sinusoidal excitations such as PWM, rectangular, and trapezoidal waveforms, the improved generalized Steinmetz equation (iGSE) predicts core losses in amorphous/nanocrystalline cores by accounting for the instantaneous rate of change of magnetic flux and the flux swing [110]. Experimental studies on nanocrystalline and amorphous cores have demonstrated that such models can capture loss variations under rectangular or converter-type non-sinusoidal excitations. Nevertheless, material-specific parameter models are still required to account for the combined effects of PWM high-order harmonics, DC bias, temperature rise, and local saturation [111].

In addition to electromagnetic excitation, mechanical constraints during actual service also significantly affect the performance of amorphous alloys, whose magnetostriction effect is more pronounced than that of silicon steel. A study of a 50 kVA three-dimensional wound amorphous alloy core showed that the saturation magnetostriction of the amorphous alloy was significantly higher than that of silicon steel [112], with core vibration mainly concentrated at 100 Hz and vibration energy distributed over 100–300 Hz. When a clamping pressure of 20 kPa was applied, the vibration displacement of the core limb decreased by 22.8% compared with the unclamped state. However, further increasing the clamping pressure could enhance the vibration again, exhibiting a nonlinear relationship. Moderate clamping is beneficial for suppressing magnetostriction induced vibration and reducing noise, whereas excessive clamping pressure may have the opposite effect, indicating that the mechanical design of transformer cores should be comprehensively optimized as a coupled magnetic-mechanical system.

Beyond electromagnetic and mechanical factors, temperature field distribution and local hot spots are also important operating factors in actual service. With increasing transformer miniaturization and power density, local overheating has become increasingly prominent. A study of the temperature field in a 50 kVA amorphous alloy wound-core transformer showed that [113], when a detailed two-dimensional model was compared with an equivalent model, the maximum temperatures of the low-voltage winding were 113.7 and 112.6 °C, respectively, while those of the high-voltage winding were 88.3 and 90.15 °C, respectively, with the maximum temperature error controlled within 1.0%. These results indicate that local hot spots in the windings and insulation structures may become life-limiting factors. For amorphous/nanocrystalline cores, a moderate temperature rise can promote structural relaxation, whereas sustained high temperatures may induce irreversible nanocrystal coarsening or detrimental phase precipitation, particularly in high-B_s_ systems with insufficient thermal stability. Meanwhile, the windings and adjacent oil ducts are critical regions for thermal management, and accurate identification of hot spots is directly relevant to insulation lifetime and operational reliability.

Noise is one of the most prominent user experience barriers to the widespread application of amorphous transformers. The λ_s_ of amorphous alloys is approximately 27 ppm, which is 3–5 times that of grain-oriented silicon steel, constituting the material origin of the relatively high noise level of amorphous transformers. Under harmonic and DC bias conditions [114], the noise is further aggravated. DC bias increases the asymmetry of the operating magnetic flux density, and both factors significantly increase the amplitude of magnetostriction induced vibration. From the materials perspective, amorphous alloys can be replaced by nanocrystalline alloys to reduce magnetostriction through the cancellation of positive and negative contributions, or the composition can be regulated to reduce λ_s_. From the engineering perspective, the overlap regions can be optimized, while clamping and damping can be improved to suppress vibration transmission and comprehensively reduce noise.

Transformer operating conditions impose special requirements on Fe-based amorphous/nanocrystalline alloys, including low loss under power frequency and harmonic conditions, high permeability and relatively high B_s_, low magnetostriction and low vibration noise, low stress sensitivity, good thermal stability, and controllable temperature rise during long-term operation. Therefore, material design should not focus solely on improving static B_s_ and H_c_ at room temperature, but should also evaluate the comprehensive service performance of the materials under the actual stress, temperature, harmonic, assembly, and noise conditions encountered during transformer operation.

### 6.2. Effects of Ribbon Fabrication Processes on Soft Magnetic Properties

The soft magnetic properties of amorphous/nanocrystalline ribbons are governed not only by alloy composition and subsequent heat treatment, but also by the planar flow casting process. Processing parameters determine ribbon thickness and its uniformity, surface quality, cooling rate, and the resulting as-quenched structure. Through structural inheritance, these factors can strongly influence the final soft magnetic properties after annealing.

**Figure 13:**
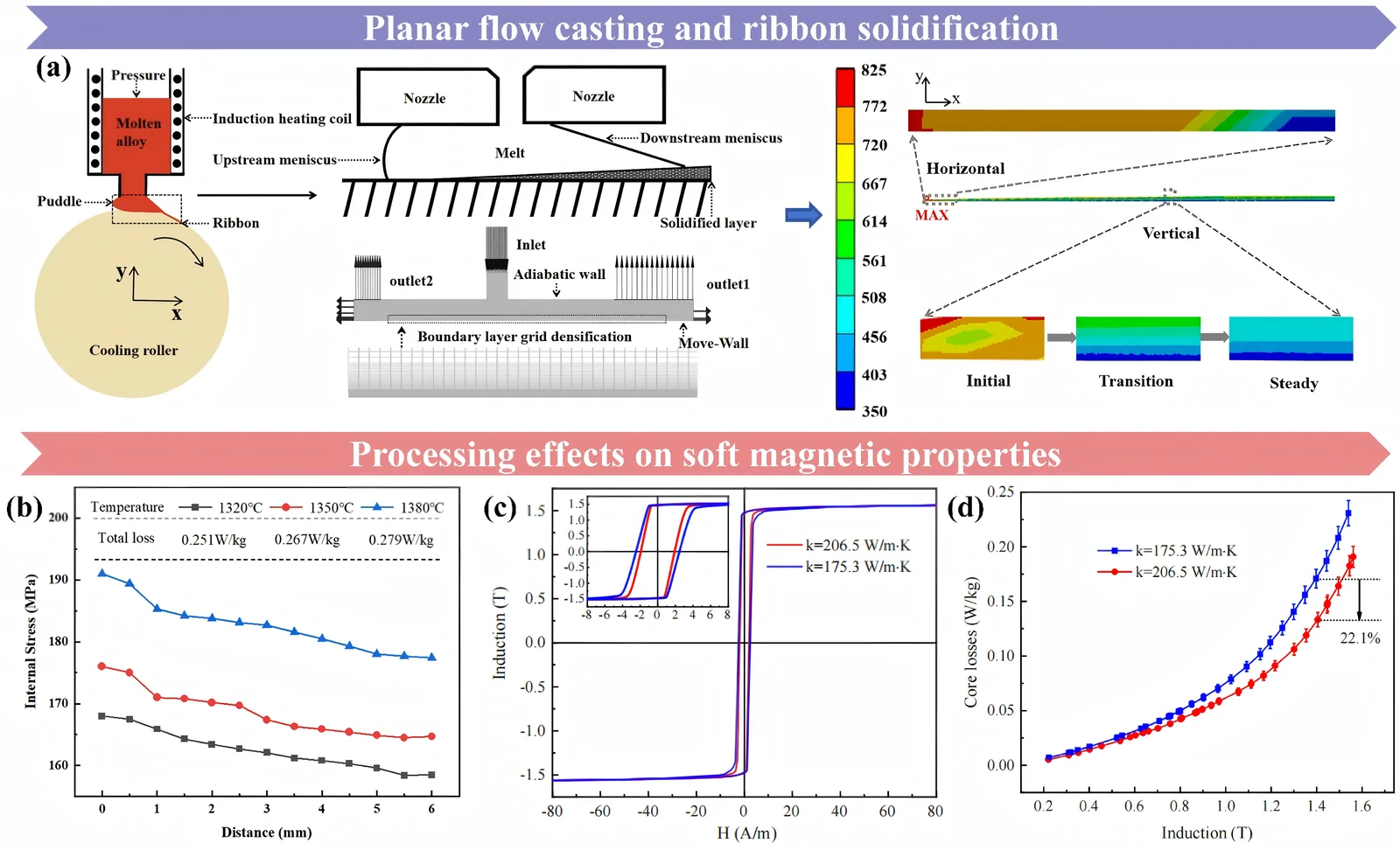
Effects of ribbon fabrication processes on the microstructure and soft magnetic properties of Fe-based amorphous/nanocrystalline alloys. (**a**) Schematic illustration of the single-roller planar flow casting process for fabricating Fe-based amorphous ribbons [116]. (**b**) Distribution of internal stress along the solidification direction and corresponding core loss variations in FeSiB amorphous ribbons at different pouring temperatures [116]. (**c**) Effect of cooling wheel thermal conductivity on the static hysteresis loops of FeSiB amorphous ribbons [118]. (**d**) Effect of cooling wheel thermal conductivity on the core loss of FeSiB amorphous ribbons [118].

The basic principle of planar flow casting (Figure 13a) is to eject the molten alloy onto a rapidly rotating copper wheel, thereby achieving rapid solidification at cooling rates of approximately 10^5^–10^6^ K/s. Key processing parameters, including wheel surface speed, injection pressure, and nozzle-to-wheel gap, jointly determine the melt-puddle geometry, heat-transfer efficiency, and ribbon thickness. Increasing the wheel speed generally produces thinner ribbons and higher cooling rates, whereas an excessively high wheel speed can destabilize the melt puddle and cause thickness fluctuations. By using cooling wheels with different thermal conductivities in combination with bottom argon purging [115], ultrathin Fe_73.5_Si_15.5_B_7_Cu_1_Nb_3_ ribbons with a thickness of only 14 μm can be fabricated, while the crystal-like locally ordered structures in the as-quenched state can also be adjusted. This indicates that the cooling process controls not only ribbon geometry but also the precursor structure that governs subsequent crystallization. The pouring temperature also af_fe_cts quenching-induced internal stress by changing the thermal conditions during solidification and subsequent contraction. As shown in Figure 13b [116], when the pouring temperature increases from 1320 to 1380 °C, the internal stress in Fe_78_Si_9_B_13_ amorphous ribbons increases overall, accompanied by an increase in core loss from 0.251 to 0.279 W/kg. These results indicate that residual stress introduced during ribbon fabrication can hinder domain-wall motion and increase magnetic loss through magnetoelastic anisotropy.

In addition to cooling-rate control, static magnetic-field-assisted melt spinning provides an additional means of controlling pre-existing nuclei without direct mechanical contact. For Fe_81.6_Si_4_B_13_Cu_1.4_ alloys [117], applying a 200 mT magnetic field along the ribbon-width direction increases the number density of α-_Fe_ nuclei in the as-quenched state from 1.1 × 10^22^ to 8.3 × 10^22^ m^−3^, while reducing their average size from 7.9 to 5.9 nm. The higher nucleation density promotes competitive growth during subsequent annealing, thereby improving microstructural uniformity and magnetic softness. Ribbon-thickness uniformity also affects the magnetic properties through both the stacking factor and structural inheritance. Thickness variations reduce the effective permeability and, more importantly, correspond to different local cooling rates, leading to variations in as-quenched free volume, medium-range order, and the density of pre-existing nuclei. These differences are inherited by the annealed nanocrystalline structure, resulting in spatial variations in magnetic properties within the core and potentially causing the overall core loss to exceed that measured for individual ribbon specimens.

Lower cooling rates can promote the formation of more crystal-like locally ordered structures in the as-quenched state and suppress nanocrystal growth during subsequent transverse magnetic-field annealing. After annealing, the 14 μm ribbon exhibits an average grain size of approximately 10 nm, together with finer magnetic domains and lower induced anisotropy. These structural differences are ultimately reflected in the magnetic losses. At 0.2 T and 100 kHz, core loss values of cores fabricated from 14, 16, 18, and 24 μm ribbons are 94, 114, 141, and 178 kW/m^3^, respectively. Therefore, the reduction in high-frequency loss achieved by decreasing ribbon thickness arises not only from the shorter eddy-current path length, but also from favorable precursor structures and enhanced grain refinement.

The thermal properties of the cooling wheel primarily affect the amorphous structure by modifying the overall thermal history during solidification. When the thermal conductivity of the cooling wheel used for Fe80Si9B11 ribbons increases from 175.3 to 206.5 W/m·K [118], the cooling rate increases from 1.2 × 10^6^ to 1.44 × 10^6^ K/s. Ribbons produced using the higher-thermal-conductivity cooling wheel exhibit narrower hysteresis loops (Figure 13c). After annealing, H_c_ decreases from 2.48 to 1.92 A/m, while core loss at 1.4 T and 50 Hz decreases from 0.1708 to 0.1331 W/kg, corresponding to a reduction of 22.1% (Figure 13d). However, excessively high cooling rates may increase quenching-induced stress and structural inhomogeneity, and the optimal cooling range depends on the GFA and crystallization kinetics of the alloy. Surface quality is also an important factor. Owing to differences in interfacial heat transfer and solidification conditions, the wheel-contact surface and free surface can exhibit different roughness and local crystallization states. The effect of surface pre-crystallization is determined not simply by whether crystals are present, but by the size and spacing of the pre-existing crystals and by the alloy’s ability to suppress subsequent grain growth. When elements such as Nb and Mo inhibit grain-boundary migration, high-density pre-existing crystals with spacings on the order of tens of nanometers can promote uniform fine crystallization through competitive growth. In contrast, coarse or sparsely distributed local crystals, together with nonuniform crystallization associated with surface protrusions, oxidation, or inclusions, can generate local stress concentrations and domain-wall pinning and may promote abnormal grain growth during subsequent annealing.

The processing atmosphere mainly affects the tendency for surface crystallization through changes in surface chemistry. Under a low oxygen partial pressure, Ti impurities tend to segregate to the free surface and undergo selective oxidation, forming TiOx heterogeneous nucleation sites [119]. When the oxygen partial pressure is increased to 10 kPa, a relatively uniform FeSiBPC-O oxide film forms and suppresses Ti segregation and surface crystallization. The subsequent effect of the surface-crystallized layer depends on whether its thickness and grain size fall within a range favorable for competitive growth.

The fabrication of high-quality amorphous ribbons therefore depends not only on alloy composition but also on systematic control of the entire casting process, including the protective atmosphere, nozzle material, cooling-wheel condition, and related processing parameters. Pre-existing α-Fe crystals, crystal-like clusters, surface oxides, and thickness variations introduced during ribbon fabrication can all influence the grain size, magnetic domain structure, permeability, and core loss after annealing. Thus, precise control of the ribbon-fabrication process provides the essential processing foundation for translating favorable intrinsic material properties into high-performance magnetic cores.

### 6.3. Wound-Core Forming and Impregnation Processes

Amorphous/nanocrystalline transformer cores are typically manufactured through a sequence of processes including ribbon winding, annealing, impregnation and curing, and cutting and jointing [120]. Within this manufacturing chain, mechanical stresses introduced during winding and additional stresses generated during impregnation and curing are key factors responsible for the deviation of finished-core performance from the intrinsic properties of the ribbon material [121]. Understanding the effects of mechanical stress on the soft magnetic properties of amorphous/nanocrystalline alloys and developing effective stress-mitigation strategies are therefore essential for translating favorable intrinsic material properties into high-performance transformer cores.

**Figure 14:**
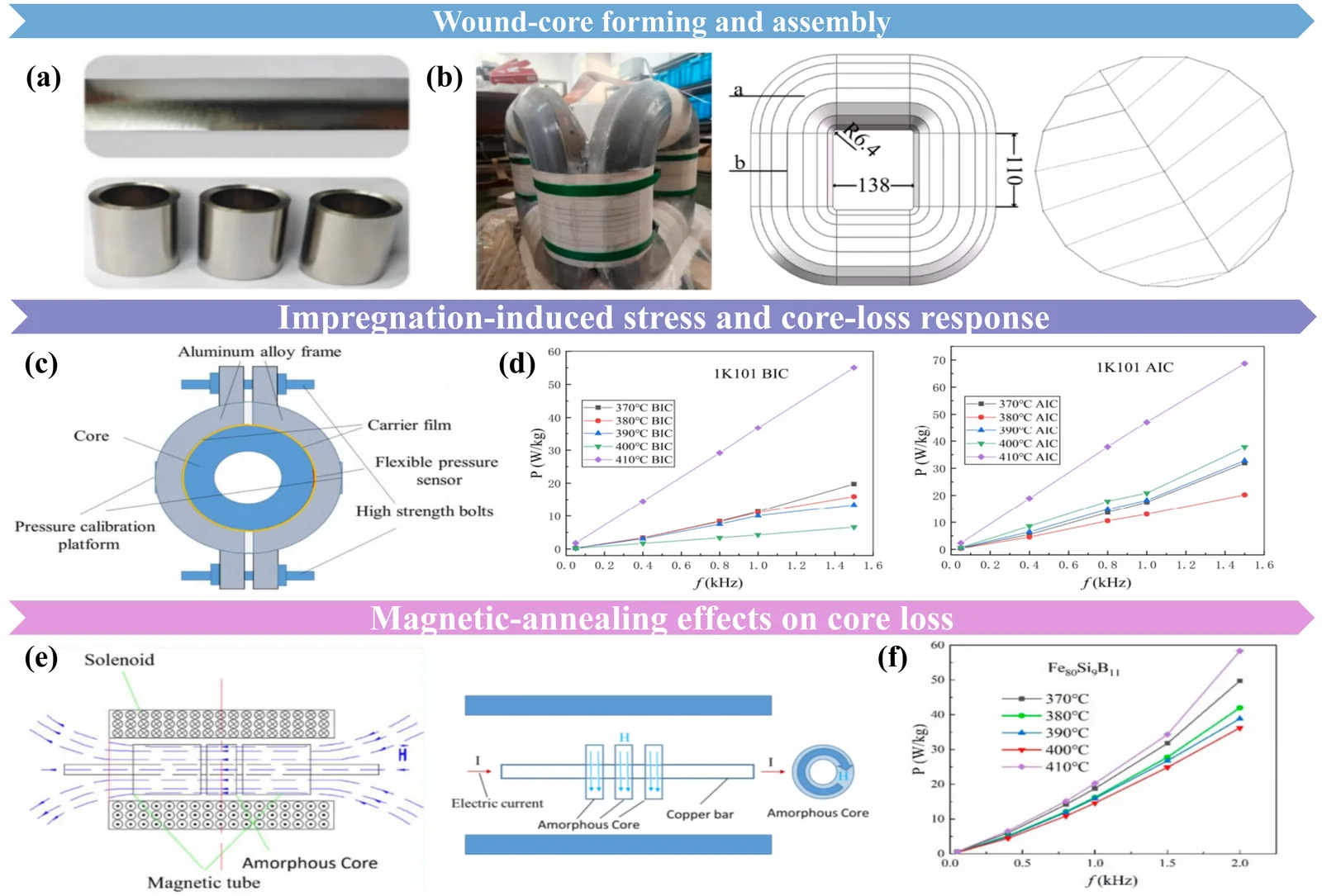
Photographs, finite element model, and magnetic properties of Fe-based amorphous wound cores. (**a**) Photographs of Fe-based amorphous ribbons and the resulting wound toroidal core [121]. (**b**) Photograph, finite element model, and core limb cross-section of a three-dimensional wound Fe-based amorphous alloy core, points a and b indicate the selected locations for vibration analysis. [113]. (**c**) Schematic illustration of the loading and testing apparatus for simulating radial compressive stress in interference-fitted wound cores [125]. (**d**) Variation of P with frequency for 1K101 amorphous wound cores before and after impregnation and curing at different annealing temperatures [125]. (**e**) Schematic illustration of transverse and longitudinal magnetic field annealing of amorphous wound cores [128]. (**f**) Variation of P with frequency for FeSiB amorphous wound cores at different annealing temperatures [128].

The detrimental effects of forming-induced stress in amorphous/nanocrystalline cores originate from their pronounced magnetoelastic coupling. During winding, a stress gradient develops through the ribbon thickness, with the inner surface subjected to compression and the outer surface to tension. For amorphous alloys with positive magnetostriction, tensile and compressive stresses induce easy magnetization axes parallel and perpendicular to the stress direction, respectively, resulting in nonuniform magnetic anisotropy and distorted magnetic domain structures. Macroscopically, these effects lead to increased coercivity and reduced permeability. Accordingly, H_c_ of a wound core is generally higher than that of the corresponding annealed ribbon, accompanied by increased magnetic loss [122]. Fe-based amorphous/nanocrystalline ribbons can be wound into toroidal cores (Figure 14a) and further assembled into three-dimensional wound-core structures [121], for which finite-element models and core-limb cross-sections can be established (Figure 14b). The multi-frame configuration and local bending regions inevitably introduce bending and assembly stresses during core fabrication [113].

When 17–27 μm amorphous ribbons are fabricated into double-U cut cores for 230 V, 110 VA transformers, core loss is only 0.59 W, more than 87% lower than that of silicon-steel cores, although the total mass increases from 1.97 to 2.21 kg [123]. A major challenge in large wound-core fabrication is to balance magnetic-path continuity with manufacturing feasibility. The ribbons must first be stacked into multilayer strips, followed by fixed-length cutting, stepped lapping, forming, and magnetic-field annealing [124]. Because of their small thickness, low stiffness, and high stress sensitivity, these ribbons are difficult to cut, stack, and clamp. The fabrication process must simultaneously ensure magnetic-path continuity in the lap-joint region and a high stacking factor while avoiding additional losses caused by excessive bending and clamping. During core assembly, radial clamping or interference fitting can generate substantial local compressive stresses, as illustrated by the compressive-stress loading device in Figure 14c. Such compressive stresses modify the magnetoelastic anisotropy of the alloy and consequently deteriorate its magnetization behavior and loss performance [125].

Impregnation and curing constitute another critical step in fixing and mechanically stabilizing the magnetic core. The main challenge arises from the nonuniform stress distribution generated by resin penetration and curing shrinkage. Figure 14d directly compares the losses of 1K101 wound cores before impregnation and curing (BIC) and after impregnation and curing (AIC). After impregnation, the core loss increases overall, and the annealing temperature corresponding to the minimum loss shifts, indicating that stresses generated by resin curing disturb the previously optimized magnetic state. Helbling et al. [126] showed that, in an industrial process involving two vacuum-impregnation cycles and two thermal-curing cycles, both resin deposition on the outer core surface and penetration into interlayer gaps alter the magnetization curve and total loss. Greater resin penetration between ribbon layers results in more pronounced degradation of the magnetic properties. Comparative experiments on different resin-deposition configurations further demonstrated the importance of core orientation during impregnation [127]. Horizontally positioned cores develop a thicker surrounding resin layer, whereas vertically positioned cores exhibit only localized resin accumulation near the suspension point. At 50 Hz, the increase in loss for horizontally impregnated cores is approximately five times that observed for vertically impregnated cores. These results indicate that core orientation during impregnation directly affects resin flow and final resin distribution. The resulting nonuniform resin distribution produces a correspondingly nonuniform local stress field, whose detrimental effect on magnetic properties can be more severe than that caused by a uniform increase in average stress and is also more difficult to predict and control.

Impregnation and curing not only increase core loss but can also shift the optimal annealing window of wound cores. Taking Fe_80_Si_9_B_11_ amorphous cores as an example [125], core loss can be as low as 6.6 W/kg at 1.2 T and 1.5 kHz after annealing at 400 °C before impregnation. After impregnation and curing, however, core loss increases to 20.2 W/kg under the same conditions, and the optimal annealing temperature shifts to 380 °C. This result indicates that impregnation-induced stress modifies the magnetic state established during annealing. Therefore, the annealing schedule should be designed in conjunction with the subsequent impregnation process, and the two should be optimized as a coupled processing sequence. An annealing condition selected solely on the basis of pre-impregnation performance cannot ensure that the finished core will retain the minimum loss. To further alleviate residual stresses introduced during winding, assembly, and impregnation, magnetic-field annealing can be employed. As shown in Figure 14e [128], transverse and longitudinal magnetic-field annealing can induce magnetic anisotropy along different directions while promoting stress relaxation and magnetic-domain rearrangement. Figure 14f further shows that the core loss of Fe_80_Si_9_B_11_ wound cores varies markedly with annealing temperature, with a distinct optimal temperature window. This confirms that appropriately designed annealing is an effective means of reducing forming-induced residual stress and restoring low-loss soft magnetic behavior. Studies of interfacial wettability have also revealed a direct relationship between stress state and resin wetting behavior [129]. For stress-annealed FeNi-based amorphous surfaces, the epoxy-resin contact angle increases approximately linearly with annealing tensile stress, from about 36.5° to 44.7°, with a fitted slope of approximately 0.035°/MPa. This indicates that stress-induced structural anisotropy can simultaneously modify magnetic anisotropy and resin wettability, thereby influencing resin penetration depth and distribution uniformity during impregnation.

Various strategies have been developed to mitigate stresses introduced during impregnation and curing. In a typical impregnation process, the annealed core is immersed in an epoxy- or silicone-based resin, subjected to vacuum impregnation, and subsequently thermally cured to bond the ribbon layers into a mechanically stable structure. However, volumetric shrinkage during resin curing generates substantial interlayer shrinkage stress, while differences in thermal expansion coefficients between the resin and metallic ribbon produce additional thermal stresses during temperature variations. Through magnetoelastic coupling, these stresses reintroduce magnetic anisotropy and can partially offset the improvements achieved by annealing. Current approaches include the use of low-shrinkage, low-modulus flexible resin systems to reduce stress transfer, optimization of the curing-temperature profile to minimize thermal stress, and introduction of stress-buffer layers on the ribbon surface to interrupt stress transmission. In addition, dry-fixation methods that avoid resin impregnation, such as elastic binding tapes and flexible clamps, have recently been adopted in some products. These approaches eliminate impregnation-induced stress at its source, although their mechanical stability and long-term reliability still require further validation.

## 7. Conclusions and Outlook

Fe-based amorphous/nanocrystalline alloys have emerged as important candidate materials for high-efficiency transformer cores because of their low H_c_, high permeability, and low core loss. Focusing on key issues such as high-frequency loss, the upper limit of B_s_ in the amorphous state, exchange averaging of random anisotropy, the evolution of alloy systems, and heat-treatment control, a multilevel relationship among composition, microstructure, processing, and soft magnetic properties has gradually been established. The low magnetocrystalline anisotropy and high electrical ρ arising from the long-range disordered amorphous structure provide the intrinsic basis for low loss at high frequencies, while exchange averaging of random anisotropy through intergranular exchange coupling after nanocrystallization underpins the excellent soft magnetic properties of nanocrystalline alloys. The evolution of four representative alloy systems demonstrates that compositional design has progressed from the pursuit of extremely low H_c_ toward multidimensional optimization involving high magnetic flux density, high-temperature stability, and cost effectiveness. Metalloid and transition-metal elements contribute to microstructural refinement by adjusting GFA, promoting nucleation, and suppressing grain growth. The four representative heat-treatment routes further improve the overall magnetic performance through coordinated control of structural relaxation, crystallization kinetics, and magnetic anisotropy. In addition, various amorphous/nanocrystalline composite structures provide further opportunities for jointly optimizing microstructure and magnetic properties.

For transformer applications, material evaluation needs to move beyond individual performance indicators measured under laboratory conditions toward a comprehensive assessment covering materials, processing, core fabrication, and service conditions. Non-sinusoidal excitation, DC bias, mechanical constraints, and local temperature rise can alter the translation of intrinsic material properties into device-level performance, while residual stresses introduced during winding, annealing, and impregnation may shift previously optimized magnetic states. Future research should therefore focus not only on achieving higher B_s_ or lower H_c_, but also on improving the consistency of high-frequency loss, the stability of continuous ribbon manufacturing, the robustness of heat-treatment processes, and performance evaluation under practical operating conditions. For high-frequency performance and scalable manufacturing, recent industrial-scale Fe-rich ribbons provide useful reference points: a core loss of approximately 75 W/kg at 10 kHz and 1 T, together with a ribbon width of 60 mm and a thickness of 25 ± 1 μm, has been demonstrated [28]. Accordingly, ≤75 W/kg and ≥60 mm are adopted here as literature-supported reference benchmarks rather than universal requirements. For engineering robustness, limiting transverse thickness variation to ±5% and the increase in core loss after winding, assembly, and impregnation to <10% are proposed as process-control targets. Since the effective annealing window of current high B_s_ nanocrystalline alloys can be only ~20–30 K, extending the stable window to >50 K while maintaining variations in H_c_ or core loss within 10% is proposed as a prospective engineering target for improving batch-process stability [130]. For performance evaluation, standardized testing should cover sinusoidal and non-sinusoidal excitation, with and without DC bias, room and elevated temperatures, and unconstrained and assembled states. Frequency, peak magnetic flux density, waveform, DC bias, temperature, and stress state should be reported consistently. Ring specimens may be used for baseline characterization and actual transformer cores for validation, following relevant standards such as IEC 60404-6. Reference samples, benchmark curves, and standardized reporting templates should also be developed, while repeatability and reproducibility should be assessed through interlaboratory comparisons.

## Figures and Tables

**Table 1:** Effects of alloying elements in Fe-based amorphous/nanocrystalline alloys.

Element	Primary Role	Representative Beneficial Range *	Adverse Effects of Excessive Addition
Si	Inhibits α-Fe formation; enhances GFA	1–5 at.%	Magnetic dilution; reduced B_s_
C	Enhances GFA; promotes stress relaxation	2–6 at.%	Disrupts multicomponent alloy balance; property degradation
B	Regulates short-range order and M_s_	10–30 at.%	Precipitation of detrimental phases; M_s_ fluctuations
P	Stabilizes the amorphous phase; refines grains	4–8 at.%	Imbalance between thermal and magnetic properties
Ge	Broadens the annealing window	~2 at.%	Fe dilution; reduced magnetic performance gain
Co	Enhances exchange coupling	~6 at.%	Reduced magnetic moment; increased stress/anisotropy
Cu	Promotes nucleation	0.5–1.25 at.%	Excessive incorporation into crystal nuclei; reduced GFA
Nb	Suppresses excessive grain growth	0.5–2.0 at.%	Magnetic dilution; weakened intergranular isolation
Mo	Suppresses detrimental phases	1 at.%	Magnetic dilution; excessive suppression of crystallization

* The listed contents/ranges are representative rather than universal and depend on the base alloy composition, elemental interactions, and processing conditions.

**Table 2:** Comparison of Four Heat-Treatment Methods for Fe-Based Amorphous/Nanocrystalline Alloy.

	Conventional Annealing	Stress Annealing	Magnetic Field Annealing	Rapid Annealing
**Main advantages**	Simple, reproducible, and scalable	Controls magnetic domains/anisotropy	Strongly lowers Hc; effective at weak fields	Refines grains; shortens processing time
**Limitations**	over-annealing causes grain coarsening/deleterious-phase precipitation	Uniform stress and coordinated control are difficult	Requires an optimal field-temperature window; limited uniformity in complex shapes	Demands high temperature uniformity/control precision
**Industrial maturity**	Most industrially mature	Mainly laboratory-scale; continuous-processing potential	Widely validated; scale-up requires optimization	Advancing from laboratory to pilot scale
**Representative temperature/time** *	Near/above T_x_ and below T_x2_; 30–330 min	~633 K; 20 min	633–673 K; 10–60 min	753–813 K; 3–30 s

* Temperature/time values are indicative and should be adjusted relative to the alloy-specific T_x_, considering composition, ribbon thickness, and required anisotropy.

## Data Availability

No new data were created or analyzed in this study. Data sharing is not applicable to this article.

## Abbreviations

The following abbreviations are used in this manuscript:

B_s_	Saturation magnetic flux density
H_c_	Coercivity
ρ	Resistivity
λ_s_	Magnetostriction coefficient
M_s_	Saturation magnetization
GFA	Glass-forming ability
D_max_	Maximum casting diameter
T_x_	Crystallization temperature
T_x2_	Second crystallization temperature
T_c_	Curie temperature
μ	Permeability
μ_e_	Effective permeability
μ_i_	Initial permeability
DC	Direct current
